# ﻿Architecture, construction, retention, and repair of faecal shields in three tribes of tortoise beetles (Coleoptera, Chrysomelidae, Cassidinae: Cassidini, Mesomphaliini, Spilophorini)

**DOI:** 10.3897/zookeys.1177.102600

**Published:** 2023-08-30

**Authors:** Caroline Simmrita Chaboo, Sally Adam, Kenji Nishida, Luke Schletzbaum

**Affiliations:** 1 University of Nebraska State Museum, Systematics Research Collections, W436 Nebraska Hall, University of Nebraska, Lincoln, NE, 68588-0514, USA University of Nebraska State Museum Lincoln United States of America; 2 Blommekloof, Leeukloof, Mossel Bay, South Africa Unaffiliated Leeukloof South Africa; 3 Museo de Zoología, Universidad de Costa Rica, San José, & Estación Biológica Monteverde, Monteverde, Puntarenas, Costa Rica Universidad de Costa Rica Puntarenas Costa Rica; 4 USGS National Wildlife Health Center, 6006 Schroeder Rd, Madison, WI, 53711, USA USGS National Wildlife Health Center Madison United States of America

**Keywords:** Behaviour, *Calyptocephala*, camouflage, *Cassida*, debris-carrying, exuviae, faeces, pupae

## Abstract

Animal constructions are the outcomes of complex evolutionary, behavioural, and ecological forces. A brief review of diverse animal builders, the materials used, and the functions they provide their builders is provided to develop approaches to studying faecal-based constructions and faecal-carrying in leaf beetles (Coleoptera: Chrysomelidae). Field studies, rearing, dissections, photography, and films document shields constructed by larvae in two species in two tribes of the subfamily Cassidinae, *Calyptocephalaattenuata* (Spaeth, 1919) (Spilophorini), and *Cassidasphaerula* Boheman, 1853 (Cassidini). Natural history notes on an undetermined Cassidini species and *Stolascucullata* (Boheman, 1862) (Tribe Mesomphaliini) outline the life cycle of tortoise beetles and explain terms. Commonly, the cassidine shield comprises exuviae onto which faeces are daubed, producing a pyramidal-shaped shield that can cover most of the body (up to the pronotum). In *Cal.attenuata* the larval shield comprises only exuviae, while in *Cass.sphaerula*, instar 1 initiates the shield by extending its telescopic anus to apply its own faeces onto its paired caudal processes; at each moult the exuvia is pushed to the caudal process base but remains attached, then more faeces are applied over it. The larva’s telescopic anus is the only tool used to build and repair the shield, not mouthparts or legs, and it also applies chemicals to the shield. Pupae in *Cal.attenuata* retain part of the exuviae-only shield of instar VI, while pupae in *Cass.sphaerula* retain either the entire 5^th^ instar larval shield (faeces + all exuviae) or only the 5^th^ larval exuvia. The caudal processes are crucial to shield construction, shield retention on the body, and as materials of the central scaffold of the structure. They also move the shield, though the muscular mechanism is not known. Altogether the faecal + exuviae shields may represent a unique morpho-behavioural synapomorphy for the crown-clade Cassidinae (10 tribes, ~ 2669 species) and may have been a key innovation in subsequent radiation. Defensive shields and domiciles may help explain the uneven radiation of chrysomelid subfamilial and tribal clades.

## ﻿Introduction

Animal constructions have fascinated humans for centuries (Smeathman 1781), perhaps as building is one hallmark for our own genus, *Homo* L. (Hominidae). Coral reefs, beaver dams, bird nests, and spider webs are familiar structures, long attracting research attention (von Frisch 1974). The size of animal constructions ranges from microscopic diatoms to coral reef formations visible from space; between their dams and lodges, beavers (Rodentia: *Castor* L.) construct the largest mammalian constructions (Larsen et al. 2021). Animals build with many endogenous and/or exogenous materials secreted or excreted by the maker, taken from other animals, or gathered from the environment. For example, silk is the most renowned animal fibre and is produced only by arthropods; it is very versatile, in cocoons, webs, and for knitting other materials together. Silk is even secondarily co-opted by other animals, including by humans. A bird’s nest may be constructed from exogenous materials (e.g., plants, spider webs), lined with feathers (endogenous), or comprise salivary secretions (endogenous) as in nests of swifts (Aves: Apodidae) which humans eat as the birds’ nest soup delicacy (Hobbs 2004; Marcone 2005). Constructions may be fashioned by an individual or a community to serve diverse purposes—nurseries and homes, traps, pantry, defences, dispersal devices, to mark territory, to aid communication (e.g., sexual and courtship displays), as physical and chemical barriers to deter predation and parasitism, or as camouflage to sneak up on prey (Hansell 2005). Constructions may be built to withstand wind, tide, and rain and some provide thermoregulation with air-conditioning. In the marine environment, decorator crabs (Hultgren and Stachowicz 2009), sea urchins (Ziegenhorn 2017) and sand mason worms (Carey 1987) build structures for camouflage, defence, and dwelling. A few books offer a primer into the diversity, roles, and engineering skills of animal architects (e.g., McCook 1907; von Frisch 1974; Hansell 1984, 2005, 2007; Turner 2000; Gould and Gould 2007; Arndt 2013). There are also children’s book on this topic (Hutchins 1959; Dewey 1991; Nicholson 2003; Nassar and Blasco 2015; Butterfield and Hutchinson 2017). Building behaviours overlap with self-decoration behaviours where animals accumulate diverse debris on their body (see review of Ruxton and Stevens 2015).

This paper concerns building behaviours and structures of certain beetles (Coleoptera: Chrysomelidae). As context for our study, we briefly review animal builders to understand the range of study, research approaches, and implications of materials and architecture. Constructions are the outcomes of complex evolutionary, behavioural, and ecological forces. In his chapter on “Instinct”, Darwin (1859: 247–256) discussed these elements in his experiments and analyses of “cell-making insect in the Hive-bee”. His approach remains valid today: observe building repertoires, design elements, materials, and purposes. Comparative multi-level analyses of physiology, ecology, ontogeny, and history are required to understand these remarkable morpho-behavioural complexes. Constructions are rich opportunities to investigate the “extended phenotypes” of their builders (Dawkins 1982).

The study of constructions is well-developed in birds, mammals, spiders, and Hymenoptera, as evidenced by documentation of specimens (i.e., in museum collections), construction behaviours, materials, terminology, and functions. The best-known insect architects are those social insects where the entire colony builds a communal “city”, Hymenoptera (ants, bees, wasps; Fabre 1915; Wheeler 1928; Sakagami and Michener 1962; Wilson 1971) and Isoptera (termites; Lüscher 1961; Krishna and Weesner 1969; Jeanne 1975; Mathews and Mathews 1978). Constructions can be prominent surface features or extend over a wide expanse and deep underground, where specialised chambers and corridors support different activities of members and enable precise control of ventilation, heat, humidity, and responses to invasions.

**Figures 1–15.**
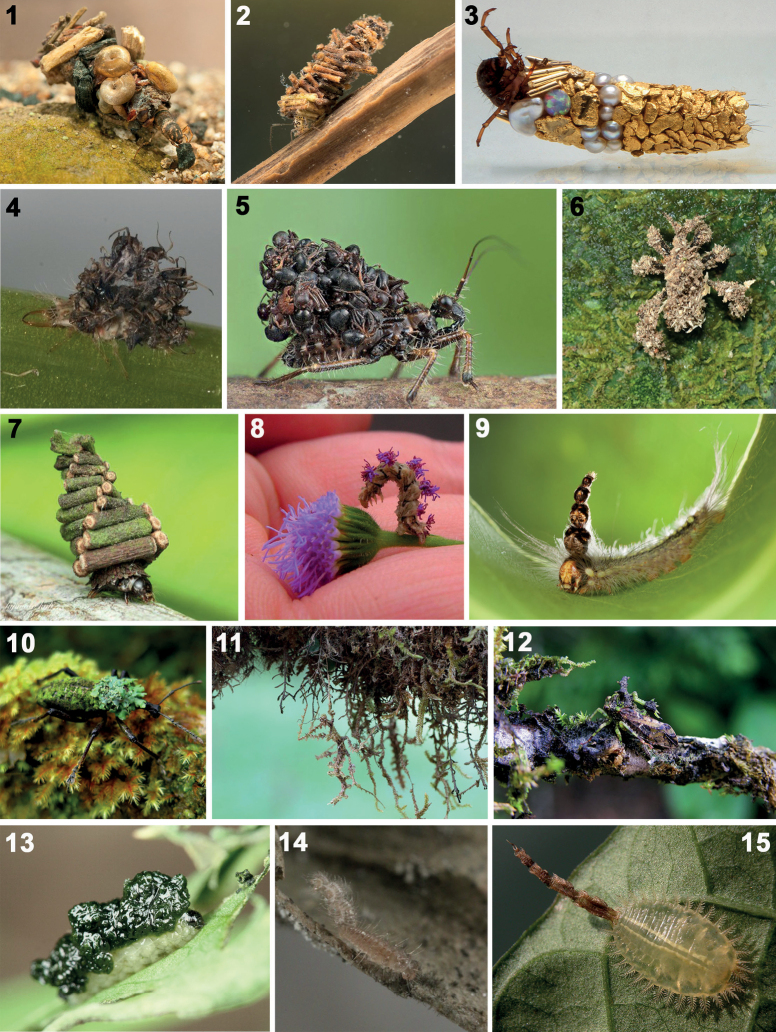
Insects with backpacks. **1**Trichoptera: Caddisfly larvae in case (photograph: S. Marshall) **2**Trichoptera: Caddisfly larvae in case (photograph: S. Marshall) **3**Trichoptera: larva with its case, 1980–1994, gold, opal, pearls (case length = 1 inch; photograph: H. Del Olmo (from Hubert Duprat exhibition, ADAGP)) **4**Neuroptera: Chrysopidae: larva with exuvial debris (photograph: Masayuki Hayashi) **5**Hemiptera: Reduviidae: assassin bug, Singapore (photograph: Nicky Bay) **6**Hemiptera: Reduviidae, assassin bug, Costa Rica (photograph: Dieter Mahsberg) **7**Lepidoptera: Psychidae: caterpillar with its bag **8**Lepidoptera: Geometridae: Wavy Emerald Moth caterpillar, *Synchloraaerata* (Fabricius, 1798), covering itself with petals of its host, *Liatris* Gaertn. ex Schreb. sp. (Asteraceae) (photograph: Hope Abrams) **9**Lepidoptera: Nolidae: caterpillar of *Urabalugens* Walker, 1866 with stack of their exuvial head capsules, Australia (photograph: Alan Henderson) **10**Coleoptera: Curculionidae, *Gymnopholus* Heller, 1901 weevil carrying lichen garden, Papua New Guinea (photograph: Adrian Tejedor) **11**Phasmida: stick insect, *Trychopepluslaciniatus* (Westwood, 1874), with exoskeleton modified to appear like moss, Costa Rica (photograph: Kenji Nishida) **12**Hemiptera: Membracidae with exoskeleton modified to appear like moss, Costa Rica (photograph: Kenji Nishida) **13**Coleoptera: Curculionidae: weevil larva retains moist faecal coat (photograph: Filip Trnka) **14**Coleoptera: Erotylidae: larva of *Toramus* Grouvelle, 1916 with shield of exuviae held on setae (photograph: Takahiro Yoshida) **15**Coleoptera: Cassidinae: Cassidini: larva of *Microctenochira* Spaeth, 1926 undetermined species with shield of exuviae only (photograph: Kenji Nishida).

Many insects are solitary architects (Figs 1–15), but they are far less known, likely due to few collections of these builders, their constructions, scant study of building repertoires, and limited evolutionary analyses. Their constructions serve most commonly to protect the vulnerable egg, larval, and pupal stages that cannot easily escape an attack. These insect mothers invest in protective devices around eggs, including elaborate oothecae (e.g., Dictyoptera: Legendre et al. 2015) and nests (e.g., mud and clay cells of some Carabidae beetles: Claassen 1919; Brandmayr and Brandmayr Zetto 1974). In Scolytidae beetles, females oviposit on or under the bark and the larvae tunnel through the wood by eating the wood and creating galleries under bark. Many insects build protections for their sedentary pupae (e.g., golden cages in Curculionidae: Hyperinae: Hoffman 1954; Scherf 1964; Janzen 1979, 1983; Aiello and Stockwell 1996). Constructions may serve as nutritional shelters, protecting the individual and providing a food source; for example, in “cigar” weevils (Curculionidae: Rhynchitinae), females roll leaves into a dual-purpose nest that serves later as a paedotrophic chamber where larvae feed on the inner walls (Brandmayr 1992).

Building materials are as diverse as the builders. Materials may be secreted by the body (endogenous), extracted from the environment (exogenous), or a combination. Endogenous secretions can create colonial structures (e.g., a coral reef) or be carried by a single individual (e.g., molluscs in their secreted shells; McDougal and Degnan 2018). Integumental secretions of slime and wax occur in sawfly larvae (Hymenoptera; Eisner 1994). Homoptera species exhibit diverse constructions: wax tail filaments (Smith 2010), sugary ‘lerp’ domiciles of scale insects (Gilby et al. 1976), and liquid marbles in aphids (Kasahara et al. 2019). Salivary secretions can serve as a glue or a building material (e.g., salivary foam moulded into pupation chambers for Criocerinae leaf beetles (Tishechkin et al. 2011). Anal secretions form the elaborate oothecae in Dictyoptera (Legendre et al. 2015).

Exogenous building materials of insects are difficult to catalogue, being so diverse, and include both organic and inorganic materials. Soil is a readily available building resource; tiger beetle larvae (Coleoptera: Cicindelidae) build burrows in the ground from where they can grab prey; some add a mud turret to raise the entrance above possible flooding (Kinsley and Pearson 1981; Shivashankar et al. 1988). Mobile residences include ornate cases by Trichoptera larvae with small pebbles or leaves (Figs 1, 2), a behaviour even co-opted for insect-built jewellery (Fig. 3; Duprat 2020). Leaves are an abundant resource; simple leaf constructions can be achieved by targeted cutting to bend over the leaf (e.g., some Lepidoptera, Loefler 1996; some cassidine beetles, Prathapan et al. 2009). Complex leaf constructions require more time (e.g., rolled leaves of Attelabidae weevils, Vanin and Bená 2020; glued leaves of some Thysanoptera (thrips), Mound and Morris 1999). Many Lepidoptera caterpillars use their silk to sew twigs (Fig. 7) or leaves into tunnels, tubes, and portable cases (e.g., Psychidae bagworms, Sharp 1899; Frowhawk 1913; Bucheli 2002). Embioptera make silken galleries where they live (Büsse et al. 2015). Exogenous materials may be harvested from the droppings of other animals; for example, mollusc shells adopted or robbed by hermit crabs (Rodrigues and Rodrigo 2009) or homopteran wax stolen by Neuroptera (Eisner and Silberglied 1988). Some constructions are compound combinations of exogenous and endogenous materials (e.g., a bird’s nest of twigs and spider silk, Hansell 2005).

Many solitary insect builders carry a ‘backpack’ with simple or compound ‘debris’ (endogenous, exogenous, environmentally acquired, organic or inorganic). Debris backpacks provide the builder with a mobile cloak that is usually assumed as a camouflage to avoid predators or a disguise for hunting (Cardé and Bell 1995; Tauber et al. 2014; Wang et al. 2016). Inorganic ‘debris cloaks’ of soil dust and small sand grains are found in insects (Odihiambo 1958; McMahan 1982, 1983a, b; Cardé and Bell 1995; Eisner 2003). In Trichoptera (caddisflies) constructed cases of silk may be decorated with sand, stones or shells and are used as retreats, homes, and to seine water for food (Wallace 1975; Wallace and Sherberger 1975; Otto and Svenson 1980; Ferry et al. 2013). The plaster bagworm (Lepidoptera: Tineidae) similarly makes a silken case that traps soil, lint, and even paint chip (Aiello 1979; Villanueva-Jimenez and Fasulo 1996). Organic debris cloaks can comprise small plant fragments such as twigs, leaves, trichomes, and wood fibres (Eisner et al. 2001). Nymphs of *Reduviuspersonatus* (L.) (Hemiptera: Reduviidae) are called “masked hunters” because of their debris of dust and soil (Fig. 6; Harz 1952; Dispons 1955; Cardé and Bell 1995; Cai et al. 2002; Weirauch 2006; Ramírez et al. 2013). Some Chrysopidae (Neuroptera) retain trichome debris covers (Smith 1922, 1923; Stitz 1931; Eisner et al. 1978, 2001; Eisner and Eisner 2000b; Anderson et al. 2003; Nakahira and Arakawa 2006; Haruyama et al. 2012). Remarkably, some insects grow a living backpack, a garden of lichens, algae, mosses, and fungi (Fig. 10; Gressitt et al. 1965, 1968). Gressitt (1977) used the term “epizoic symbiosis”; this camouflage resembles those insects that truly are morphologically adapted with a moss-like appearance that matches their lichen + moss covered habitat (e.g., Figs 11, 12).

Organic debris backpacks comprising insect exoskeletons (exuviae, cast skins) appear in diverse insects (Figs 4, 5, 9, 14, 15). These exuviae can be the builder’s own castoffs or, more macabre, from their prey. Examples of the first type, retaining their own exuviae, are exhibited in some Lepidoptera and Coleoptera larvae. An Australian caterpillar retains a stack of its previous head capsules, giving it the nickname “mad hatterpillar” (Fig. 9; Lepidoptera: Nolidae; McFarland 1980; Pearson 2013). In Coleoptera, exuvial retention by larvae is known in some Erotylidae (Figs 14; Leschen 2003; Yoshida and Leschen 2020) and in Cassidinae (Fig. 15; Chaboo 2007). The second type of exuvial retention uses those of prey and has been described as a “corpse cover” (Brandt and Mahsberg 2002), a “corpse camouflage” (Stromberg 2012), and a “wolf in sheep’s clothing” strategy (Eisner et al. 1978, after ancient rhetorical Greek and Italian fables, e.g., Basilakis in the 12^th^ century; Beneker and Gibson 2016; Abstemius 14^th^ century; the Bible (King James Bible Online 2023)). Some Chrysopidae larvae (Fig. 4; Neuroptera) carry the exuviae of their aphid prey, to fool aphid-tending ants (Hayashi and Nomura 2011). Many Hemiptera adults and nymphs retain corpse backpacks (Fig. 5; Odihiambo 1958; McMahan 1982, 1983a, b; Zeledón et al. 1973; Weirauch 2006), some adding dust and soil, for a mix of organic and inorganic debris. Corpse covers and debris cloaks may provide mechanical protection, from weather or predators (e.g., spiders, lizards), or permit aggressive mimicry towards their prey (e.g., ants, termites). Olfactory cues can mask the predator (Odihiambo 1958; Brandt and Mahsberg 2002; Jackson and Pollard 2007; Stromberg 2012) or may become a secondary signal that attracts enemies (Agelopoulus et al. 1995; Benelli et al. 2013; Huang et al. 2022).

Dung (faeces, frass, fecula) is an unconventional organic debris as faeces are typically considered unappetising and unhygienic waste products, vectors of pathogens, and an offensive by-product of animal metabolism. Most animals simply eliminate and avoid their waste, even finding creative ways to dispose of their faeces (e.g., mining insects, Frost 1942). Yet, faeces are a cost-free and readily available benefit of regular feeding. In Mammals, faecal piles function as territory markers (e.g., Stewart et al. 2001) and latrine sites (e.g., meerkats, Jordan et al. 2007). Counter-intuitively, faeces are a resource; indeed, humans have been using dung (Henry et al. 2016; Arranz-Otaegui et al. 2017; Smith et al. 2022) as a fertiliser since early agriculture, to burn as fuel, for plastering adobe walls and floors (faeces mixed with mud and twigs), in beauty facials (”Uguisu no fun”, Moore 2001) and even in ancient (Ge 2000 [4^th^ century]) and contemporary medical faecal transplants and enemas (e.g., Fecal Microbiota Transplantation or FMT; Eiseman et al. 1958; Zhang et al. 2012).

Dung beetles (Scarabaeidae) may be the most famous insects associated with faeces. Both dung beetles and burying beetles (Silphidae) use vertebrate dung for brood balls (Waterhouse 1974; Scholtz and Grebennikov 2004). Many fly groups are also renowned to use faecal habitats.

Many terms for insect faeces appear in the literature. Frost (1942) used ‘faeces, fecula and frass’ which have become widely used. Other terms are excrement (Hislop 1872; Scudder 1891; Muir and Sharp 1904; Blatchley 1924; Flinte and Valverde de Macédo 2004), excreta (Wood 1966; LeSage 1982; Jolivet and Verma 2002), and scat (Lécaillon 1896; Hinton 1981). Faeces are produced mainly by immature insects since most adult insects produce little wastes. Insect faeces can serve various purposes, such as adult aggregation, finding mates, brooding, or oviposition deterrent; they can signal pest issues. They can also recycle faeces in multiple ways; the process is sophisticated in social insects where faeces are used as a structural component of the nest and hive walls and as a substrate for growing fungi (Hansell 2005; Weiss 2006). In Coleoptera, faeces can serve for adult aggregation (Tenebrionidae: flour beetles; Suzuki 1985), to find mates (e.g., Cerambycidae: *Hylotrupesbajulus* (L.); Fettköther et al. 2000), brooding, or as an oviposition deterrent (e.g., weevils and cerambycids; Anbutsu and Togashi 2002; Addesso et al. 2007).

Insects in Coleoptera, Diptera, and Lepidoptera have evolved dung-carrying behaviours. Some Lepidoptera caterpillars retain a dry crust of their excreta (e.g., Noctuidae; Preston-Mafham and Preston-Mafham 2003: 406); others use their silk to knit their faeces into “frass chains” (resembling sticks) to build a retreat (e.g., Nymphalidae; Freitas and Oliveira 1992; Caldas 1994; Machado and Freitas 2001). Excremental cases are known in Diptera (e.g., Mycetophilidae; Holmgren 1907; Knab and van Zwaluwenburg 1918) and in Lepidoptera (e.g., Hesperiidae; Sharp 1899). Weevils (Curculionidae) exhibit diverse constructions: leaf-rollers (e.g., Attelabidae, Daanje 1975; Mathews and Mathews 1978), lichen-carriers (e.g., Fig. 13, Gressitt et al. 1965; Gressitt 1977; Jolivet 1988a), solid dung (e.g., Ceutorhynchini, Knab 1915), and liquid excremental covers (e.g., Cionini, Gonipterini; Knab 1915; Arzone and Meotto 1978; Janzen 1979, 1983; Crowson 1981; Aiello and Stockwell 1996). Other beetles may construct a faecal or faecal-fungal canopy or retreat (Leschen and Carlton 1993; Leschen 1994; Hanley 1996). It is important to note that faecal retention is most often exhibited by insect larvae and the behaviour has been interpreted mostly as armour, camouflage, or physical barrier to enemies (Weiss 2006).

Debris-carrying, including dung-carrying, is not simply just ‘carrying’ since individuals often exhibit specialised morphology associated with handling faeces (e.g., anal comb in some Lepidoptera, Frost 1919) or with retaining materials (special setation; Weirauch 2006; Skuhrovec et al. 2017) to build, carry, wear and even repair structures. Enhanced survivorship is often assumed, and in cases where tested, the adaptive value of debris such as frass and faeces has been demonstrated.

In this paper, we focus on faecal-recycling behaviours in Chrysomelidae (leaf beetles), one of the largest clades of beetles with > 40,000 species (Leschen and Beutel 2014). Chrysomelids use their faeces as a biomaterial for constructions and self-decoration behaviours that serve as defensive coats, mobile debris shields, and protective domiciles. Such faecal-based constructions appear as a striking pattern within Chrysomelidae, diagnosing some large subfamilies and appearing also in some small clades.

In general, leaf beetles exhibit diverse building behaviours, including oothecae with multi-layered colleterial secretions (e.g., some Cassidinae), faecal covers (Kalaichelvan and Verma 2000), or with stomach regurgitate (Jolivet and Verma 2002), larval galls (e.g., Sagrinae, Reid and Beatson 2019), and pupation chambers of soil, sand (e.g., some Galerucinae, Prathapan and Chaboo 2011), faeces (Cryptocephalinae; Brown and Funk 2005), or salivary ‘foam’ (e.g., some Criocerinae, Tishechkin et al. 2011). Bruchine adults build walls within seeds to inhibit fighting (Mano and Toquenaga 2008). Simple leaf shelters are made by larvae and adults of *Leptispa* Baly, 1858 (Cassidinae: Leptispini; Prathapan et al. 2009). Many chrysomelid mothers coat eggs with glandular and excremental applications, often mixed with anal and buccal secretions, and then may cover eggs further with plant pieces or oothecal membranes or faecal cases (Muir and Sharp 1904; Fiebrig 1910; Prevett 1966; Hinton 1981; Jolivet and Verma 2002; Müller and Hilker 2004).

**Figures 16–18.**
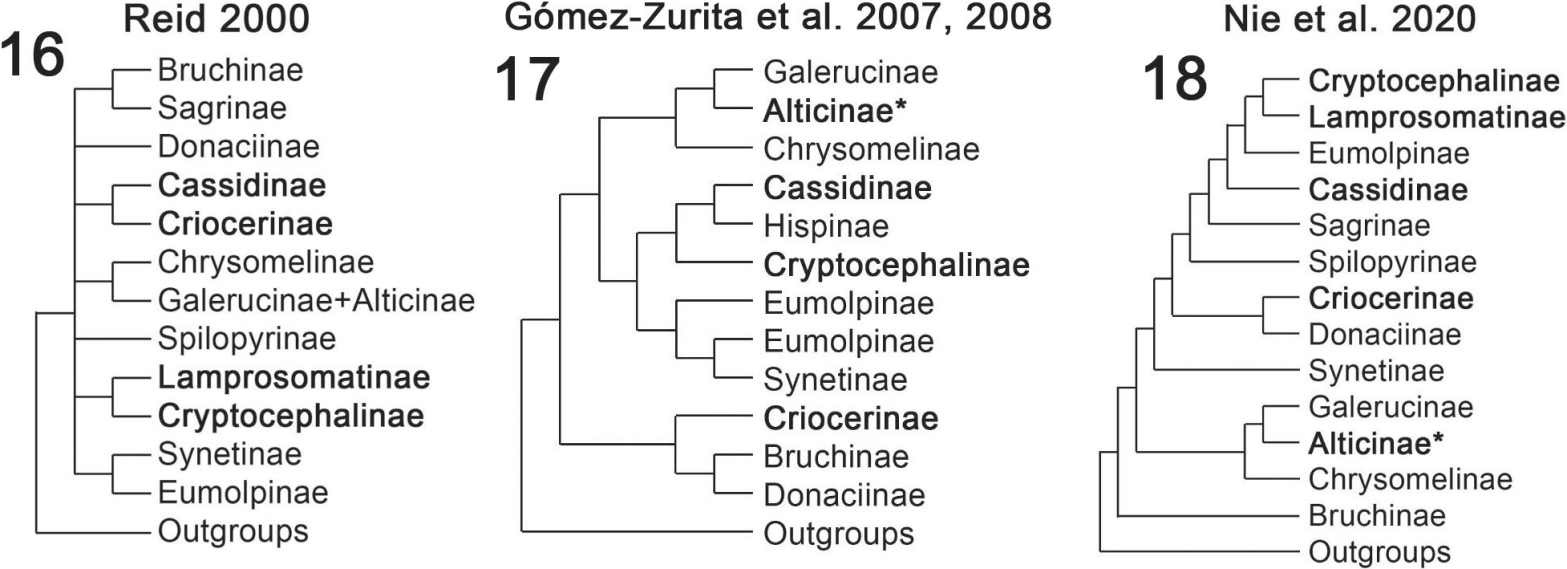
Recent phylogenetic hypotheses of Chrysomelidae subfamily relationships, redrawn by L. Schletzbaum from original sources **16**Reid (2000) (morphology-based) **17**Gómez-Zurita et al. (2007, 2008) (sequence-based) **18**Nie et al. (2020) (sequence-based). Other chrysomelid hypotheses to compare are Farrell (1998), Hunt et al. (2007), and Zhang et al. (2018, 2022). These available hypotheses are based on less than 1% taxon sampling of clade diversity. Subfamilies in bold font exhibit major patterns of faecal-based constructions. Alticinae (flea-beetles) is now regarded as the tribe Alticini within Galerucinae, so the faecal-retaining *Blepharida*-group is recognized now within Galerucinae. Only a single species in *Phola* Weise, 1890 (Chrysomelinae) has been reported to retain faeces (Chen 1964, 1985) so it is not a major pattern.

**Figures 19–26.**
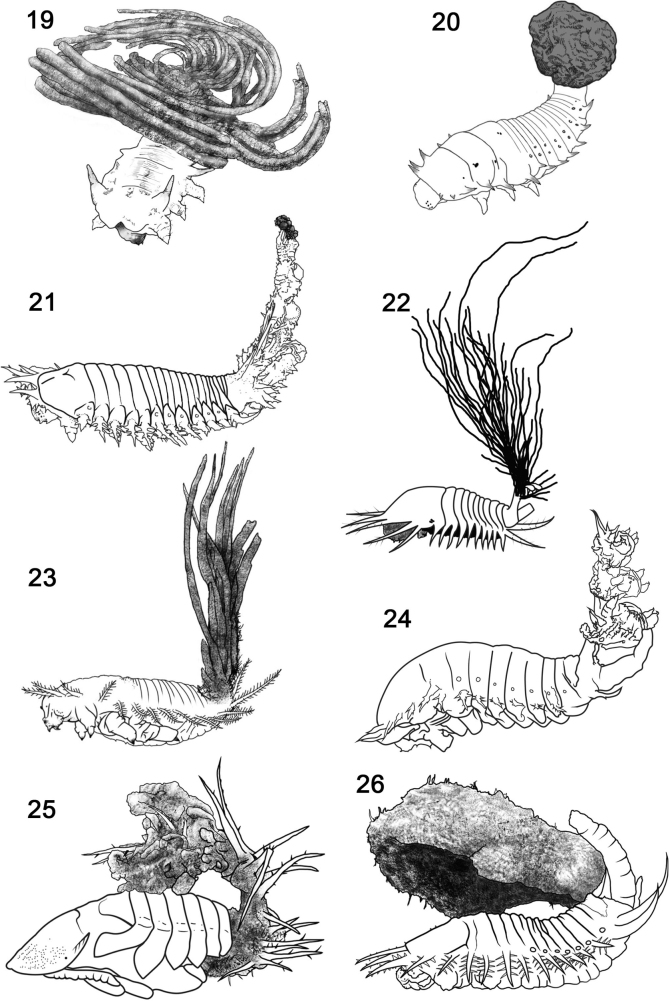
Shields of larvae and pupae in four tribes of Cassidinae (Coleoptera: Chrysomelidae) **19**Hemisphaerotini: *Hemisphaerota* Chevrolat, 1836 **20**Ischyrosonychini: *Physonota* Boheman, 1854 **21**Cassidini: *Agroiconotabivittata* (Say, 1827) **22***Aspidimorphasanctaecrucis* (Fabricius, 1792) **23**Cassidini: undetermined sp. 1 **24**Cassidini: undetermined sp. 2 from Africa, collected by C.S. Chaboo **25**Cassidini: undetermined sp. 3 pupa from Brazil, collected by D. Yanega **26**Cassidinae: Undetermined sp. 4 Costa Rica, collected by K. Nishida. Darkened sections = faeces. Redrawn by L. Schletzbaum from original sources or from specimens.

**Figures 27–34.**
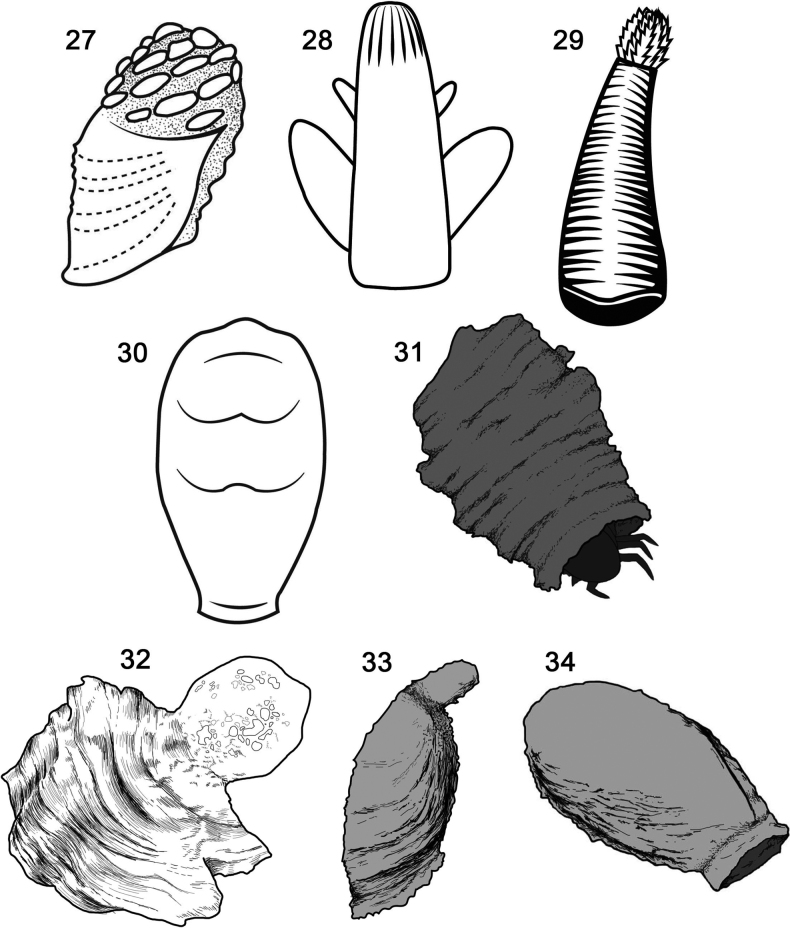
Faecal structures of larvae and pupae in Cryptocephalinae (Coleoptera: Chrysomelidae) **27***Adiscustaiwanus***28***Chlamisus* sp. 1 **29***Chlamisus* sp. **30***Coenobiustaiwanus***31***Cryptocephalustrifasciatus***32***Fulcidax***33***Neochlamisus***34**Lamprosomatinae. Redrawn by L. Schletzbaum from original sources.

**Figures 35–37.**
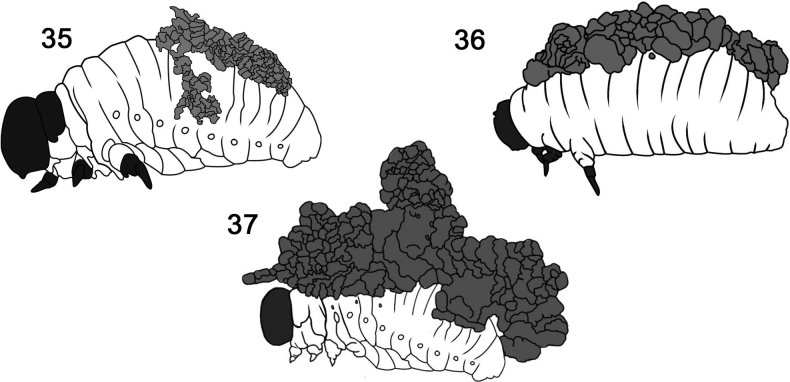
Faecal-based coats of larvae in Criocerinae (shining leafbeetles). **35**Criocerinae sp. 1 **36**Criocerinae sp. 2 **37***Lemahexastigma*. Redrawn by L. Schletzbaum from original sources.

**Figures 38–40.**
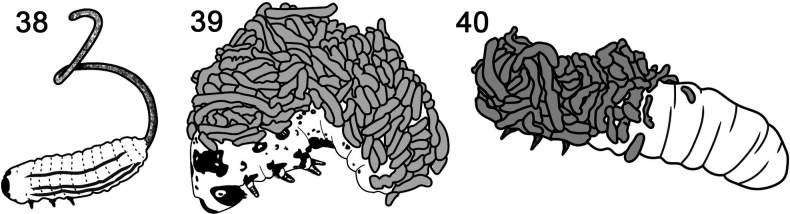
Faecal-based structures and coats maintained by larvae in the *Blepharida*-group (Galerucinae: Alticini; flea beetles). **38***Blepharidasacra***39***Diamphidia* sp. **40***Podontia* sp. Redrawn by L. Schletzbaum from original sources.

The faecal-based constructions of Chrysomelidae are not a diffuse pattern but are taxonomically focused, are ancient, dated at least 45 million years ago (Chaboo and Engel 2008; Chaboo et al. 2009), and may have three or four independent origins given simple mapping on recent phylogenetic hypotheses of the family (Figs 16–18): within the subfamily Cassidinae; the *Blepharida*-group within the subfamily Galerucinae; Criocerinae; and in the sister subfamilies Cryptocephalinae + Lamprosomatinae. Within Cassidinae (6,320 species in 37 tribes), faecal constructions diagnose a derived monophyletic clade of ten tribes (= the tortoise beetle tribes) where most larvae use their exuviae and/or faeces to build shields over the body (Figs 15, 19–26; Chaboo 2007); these shields may be retained in pupae of some species (Fig. 25). Cryptocephalinae + Lamprosomatinae (~ 6000 species) form a well-accepted clade, called Camptosomata, that is distinguished by a complex behaviour where females construct a faecal case around the single eggs and the natal larva keeps that egg case as a rigid portable home (Figs 27–34; Lawson 1976). This faecal case (= faecal bag, scatoshell) becomes the nucleus that the larva continues expanding with their own faeces; eventually the pupa inherits this construction as their pupation chamber (Brown and Funk 2005; Chaboo et al. 2016). Criocerinae is a smaller subfamily of ~ 1400 species whose larvae maintain a wet or semi-solid mass of their faeces directly on their back (Figs 35–37; Vencl et al. 2004). The *Blepharida*-group comprises ~ 21 genera (D’Alessandro and Biondi 2023) within the hyperdiverse Galerucinae (7145 species: Lingafelter and Konstantinov 2000; Nie et al. 2017); this group is distinguished by larvae that keep a single faecal strand held over the body (Fig. 38; Furth 1982, 2004; Furth and Lee 2000) or many faecal pellets directly on the dorsum (Figs 39, 40; Prathapan and Chaboo 2011; Calcetas et al. 2023). In Chrysomelinae (~ 3000 species), larval faecal tubes have been reported only in *Pholaoctodecimguttata* (Fabricius, 1775) (Chen 1964, 1985) and is a minor building pattern within this large subfamily. It is unclear at present what could be trends in innovations and maternal investments in oviposition site selection, and oothecal and egg-case construction. These chrysomelid constructions and body coats appear to be composites of endogenous and exogenous materials (Table 1), with their own faeces, exuviae, plant materials (trichomes, bark, twigs, decomposing fragments), chemical (plant or animal made), and even fungi. The endogenous materials can include faeces, anal, buccal, and other glandular products, and exuviae. The roles of each material are unknown.

Comparative surveys of the architectures of leaf-beetle constructions, detailed study of morphology associated with construction, retention and repair, and study of constructing behaviours are all needed to elucidate the apparent multiple origins and diversification of these structures. Experimental studies are needed to test proposed hypotheses about the adaptive significance of faecal-based constructions. Such data can explain if and how these unusual faecal constructions could have influenced chrysomelid diversification, producing such uneven subfamilial species diversities.

In Cassidinae (~ 6000 species), faecal-based construction behaviour is a significant macroevolutionary event with a radiation of ~ 2700 species after its origin (Chaboo 2007), assuming a single evolutionary origin of faecal shield construction. This crown-clade is called “tortoise beetles” and is distinguished by a unique combination of morphology and behaviours: the larvae are exophagous (or ectophagous) and have paired caudal processes (= urogomphi) onto which they build and carry a debris shield (Figs 15, 19–26) moulded from their own exuviae and faeces. These larvae use a telescopic anus to apply faeces to the shield. The shield can be moved over the body like an umbrella or parasol (Fiebrig 1910; Takizawa 1980; Chaboo 2007; Flinte et al. 2009; Świętojańska 2009). Some exceptions, absence of shield retention, are also known but these appear to be secondary losses given the current phylogenetic views. Tortoise beetles comprise ~ 2700 species classified into ten tribes: Basiprionotini Hincks, 1952; Cassidini Gyllenhal, 1813 (now includes Aspidimorphini and Charidotini); Dorynotini Monrós & Viana, 1949; Eugenysini Hincks, 1952; Goniocheniini Spaeth, 1942; Hemisphaerotini Monrós & Viana, 1951; Ischyrosonychini Chapuis, 1875; Omocerini Hincks, 1952; Mesomphaliini Chapuis, 1875; and Spilophorini Chapuis, 1875. The systematics of Cassidinae has been dynamic in the last 15 years and there are some disagreements on classification; we briefly discuss some issues relevant to our taxon focus in ‘Materials and methods’ below.

An obvious question is “How do tortoise beetles build their shields?” We address this specifically in three tribes Cassidini, Mesomphaliini, and Spilophorini. We aim to understand how the architecture is achieved and what morphological equipment is involved. We examine the materials, building processes, retention and repair of faecal constructions, and their inheritance from one instar to the next. Still images and short films document building behaviours and dissections help puzzle out how the materials are fitted together. We briefly review explanatory hypotheses for possible functions of cassidine shields. To date, the only study of chrysomelid faecal-constructing behaviour has been in *Neochlamisus* Karren, 1972 in the hyperdiverse subfamily Cryptocephalinae (~ 6000 spp.) by Brown and Funk (2005). Our study complements that work. Finally, we discuss the evolutionary-phylogenetic context to frame future research on chrysomelid faecal-based constructions.

## ﻿Materials and methods

We compare architectures and study construction behaviours in four species in three tortoise beetle tribes (derived Cassidinae, *sensu*Chaboo 2007) based on fieldwork in Costa Rica (2011–2021) and South Africa (2021–2022). To minimise confusion of species, we use these genus-name abbreviations: *S.cucullata* for *Stolascucullata* (Boheman, 1862) (tribe Mesomphaliini), *Cassidini* undet. sp. 4 for an unidentified species (tribe Cassidini), *Cal.attenuata* for *Calyptocephalaattenuata* (Spaeth, 1919) (tribe Spilophorini), *Cass.sphaerula* for *Cassidasphaerula* Boheman, 1854 (tribe Cassidini).

### ﻿Research approach

**Figures 41–44.**
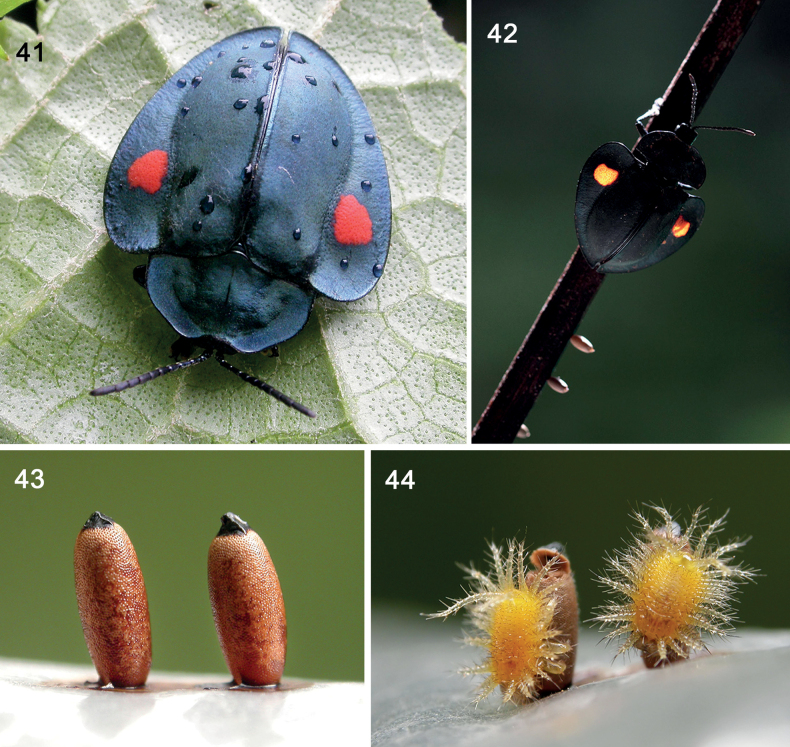
Life history in *Stolascucullata* (Boheman, 1862) (tribe Mesomphaliini) in Costa Rica **41** adult **42** female laying eggs **43** eggs, grouped but not in contact **44** neonate larvae resting on egg shell (photographs: K. Nishida).

**Figures 45–50.**
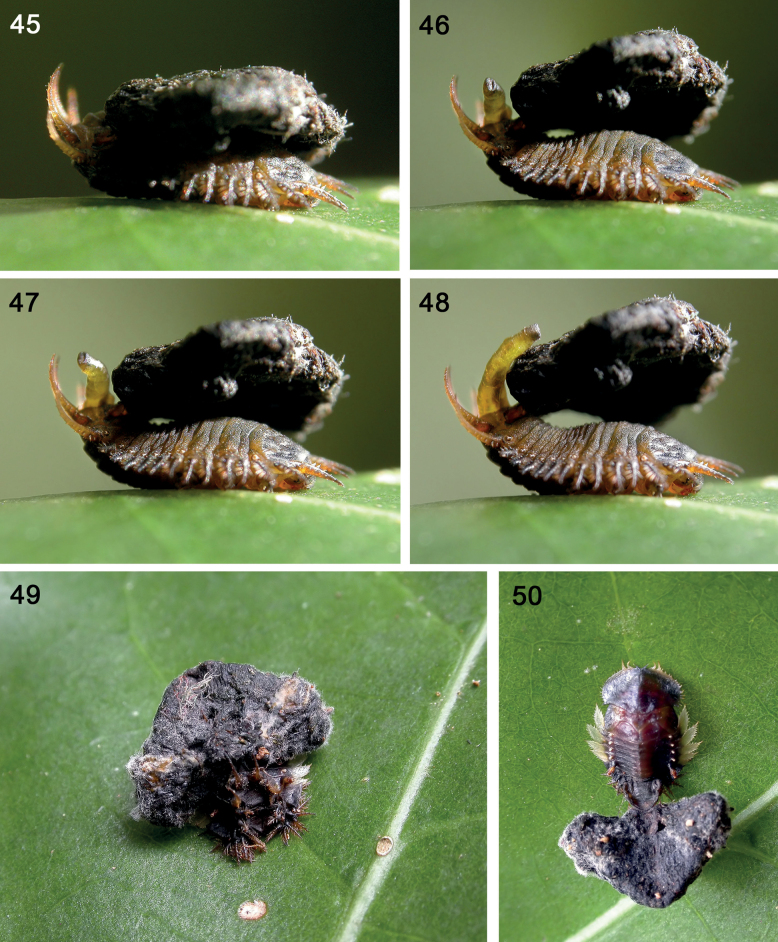
Telescopic anus and shield of larva, Cassidini undetermined sp. 4 on *Chionesylvicola* (Standl.) W. C. Burger (Rubiaceae) in Costa Rica **45–48** anus at different positions **49** pupa, postero-dorsal view **50** pupa, dorsal view (photographs: K. Nishida).

**Figures 51–58.**
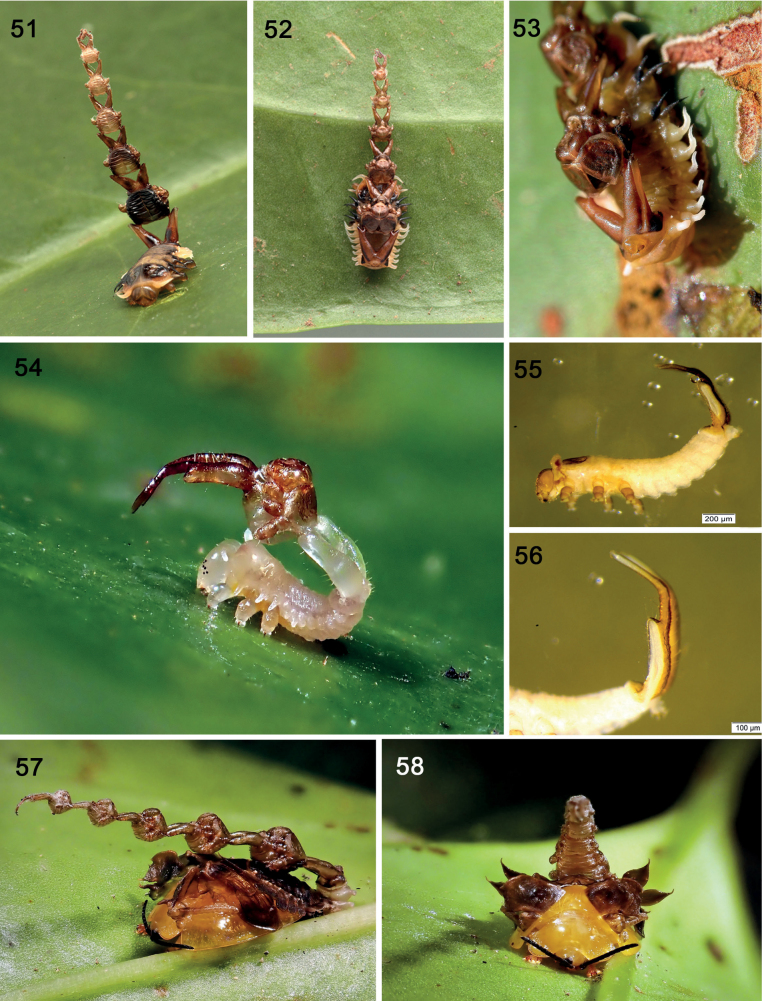
*Calyptocephalaattenuata* on the host, *Smilaxdomingensis* Willd. (Smilacaceae), Monteverde, Costa Rica **51** larva with shield of five exuviae, dating this as instar VI **52** dorsal view **53** showing exuviae folded to expose head capsule and caudal processes **54** teneral instar II larva has just exited exuvia I and is retaining it on elaborate paired caudal processes (photographs: K. Nishida) **55** instar I (~ 42 mm long), showing caudal processes **56** instar I caudal processes (photographs: CS Chaboo) **57** adult partially exiting pupal exuvia, fronto-lateral view **58** adult partially exiting pupal exuvia, frontal view (photographs: K. Nishida).

**Figures 59, 60.**
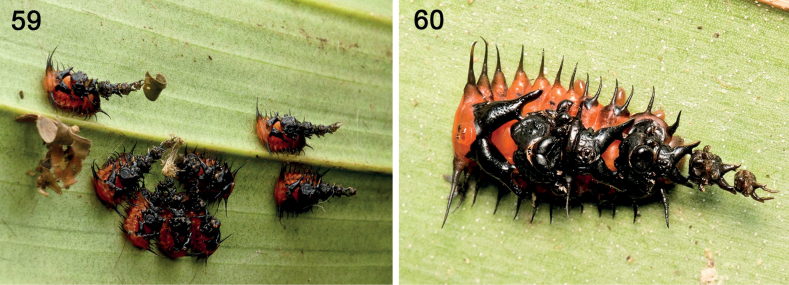
Unidentified genus, 5^th^ instar larvae of Spilophorini on orchid host in Ecuador **59** mature larvae feeding in a group; note color contrast which may be aposematic and the leaf fragment on shield of one larva **60** single larva, dorsal view, with shield of four exuviae. Note exuvial folding exposes the anus and head capsule. Bases of caudal processes are also exposed (photographs: E. Schulz).

First, we introduce concepts of life stages, structures and morphology involved in Cassidinae construction by reporting the natural history of *S.cucullata* and Cassidini undet. sp. 4 (three undet. species of Cassidini are illustrated in Figs 23–25). Second, we report on shield construction in two focal species, *Cal.attenuata* (Spilophorini) and *Cass.sphaerula* (Cassidini). Third, we compare and contrast the construction behaviours and resultant architectures, contextualising our findings within Cassidinae and Chrysomelidae. Our focal taxa here are:

1. **Tribe Mesomphaliini**: *Stolascucullata* (Boheman, 1862) (Figs 41–44). Observations, photographs, and specimen collection were made at COSTA RICA: Cartago Province, Orosi, Tapantí National Park, 9°45'38.63"N, 83°47'3.98"W, 1280 m ele., 24-vii-2011, coll. Kenji Nishida. Oviposition was observed and photographs were taken also on 31-vii-2011 by KN. The live beetles were observed in a cloud forest habitat, along an open trail. The host plant was not determined initially as the female was flying then and landed on vegetation. Later, oviposition was observed, the host plant could be identified, and the hatched larvae were followed in the field on that host plant. Identifications: there are only five or six *Stolas* Billberg, 1820 species in Costa Rica. The red marginal spot on the black elytra is found in adults of three species: one spot in *Stolascucullata* (Boheman, 1862), two spots in *Stolascostaricensis* (Champion, 1893), and two spots in *Stolaslebasii* (Boheman, 1850). Świętojańska (2009) indicated that juvenile stages are known for just five of the 187 recognised species of *Stolas*: *Stolaschalybea* (Germar, 1824), *Stolasfestiva* (Klug, 1829), *Stolasimplexa* (Boheman, 1850), *Stolaslacardairei* (Boheman, 1850), and *Stolaslineaticollis* (Boheman, 1850). Vouchers are deposited in the Museo de Zoología (MZUCR), Universidad de Costa Rica, San Pedro de Montes de Oca, Costa Rica. *Stolascucullata* was identified by CSC using the online catalogue of Borowiec and Świętojańska (2002–present). The latter indicates that the type specimen was collected by Warszewicz in Panama: Veraguas, and that Bolivia is an inaccurate locality; the type is supposed to be in the J. Weise collection, Zoologisches Museum, Humboldt Universitat, Berlin, Germany, but it cannot be located (Bernd Jaeger, pers commun.). This species is distributed in Costa Rica and Panama (Chaboo 2003). Plant: this was identified as *Neomirandeaangularis* (B.L.Rob.) (Asteraceae) by B. Haber, Monteverde. This is a new host record; Windsor et al. (1992) previously recorded *Neomirandeahomogama* (Hieron.) Rob & Brett. as a host of *S.cucullata* in Panama.
2. **Tribe Cassidini**: Cassidini undet. sp. 4 (Figs 45–50). All life stages have been documented on the host plant by KN in COSTA RICA: Puntarenas Province, Monteverde, 2016. Identifications: We await further study for more conclusive species determination. Plant: *Chionesylvicola* (Standl.) W. C. Burger (Rubiaceae) was identified by B. Haber, Monteverde. This is a new host record for Cassidinae; only six species of Cassidinae (4 Cassidini, 2 Notosacanthini) have been reported on Rubiaceae hosts (Borowiec and Świętojańska 2002–present; Monteith et al. 2021).
3. **Tribe Spilophorini**: *Calyptocephalaattenuata* (Spaeth, 1919 (Figs 51–58). Live populations were studied on four *Smilax* spp. (Smilacaceae) at COSTA RICA: Puntarenas Province, Monteverde, 1530m, 10°19'08.5"N, 84°48'32.0"W, periodically over 2014–2020, Author KN led field studies and published some natural history reports (Nishida 2014, 2015; Nishida et al. 2020). Beetles were identified by CSC. Vouchers are deposited in the Museo de Zoología (MZUCR), Universidad de Costa Rica, San Pedro de Montes de Oca, Costa Rica. The four species of *Smilax* host plants were identified by L. Ferrufino-Acosta. The life cycle of *Cal.attenuata* includes six larval instars and the pupa; all carry exuvio-faecal shields on paired caudal processes (Figs 54–57; = urogomphi). The shield is composed solely of exuviae of previous instars and no faeces. Adults exit the pupal exuvia by splitting the anterior margin of the pupa (Figs 57, 58). Interestingly, adults eclose partly but stay in situ for 2-3 days, hardening up, before exiting completely from the pupal exuvia. Photographs of juveniles (Figs 59, 60) of an unidentified third species from Ecuador were sent by photographer Eerika Schulz to author CSC in 2018 who identified the species as belonging to Spilophorini. Pedro Ríos Guayasamín and students, Universidad Estatal Amazónica, are studying this population on an Orchidaceae host, and will send specimens to CSC for identification.
4. **Tribe Cassidini**: *Cassidasphaerula* (Figs 65–89). Author SA conducted fieldwork in 2021–2022, observing populations of an endemic beetle on its host, *Arctothecaprostrata* (Salisb.) Britten (Asteraceae) in various locations around Mossel Bay, South Africa, 33°57'58"S, 22°5'24"E. Adam et al. (2022) reported on natural history. The life cycle has five larval stages, all with exuvio-faecal shields, and the pupa that may carry shields of exuviae only or shields of exuviae and faeces. **Identifications.** Beetles were identified by CSC and confirmed by E. Grobbelaar. **Vouchers.** These are deposited at South Africa National Insects Collection (SANBI) and loaned to CSC.

### ﻿Permits

Resolutions # 039-2013-SINAC; # 080-2013-SINAC; SINAC-SE-GASP-PI-R-058-2014 (3 total) were issued by Ministerio de Ambiente y Energia (MINAE), Costa Rica. These allowed research/collecting and specimen export. Permits were issued by Sistema Nacional de Áreas de Conservación, Ministerio de Ambiente y Energía (MINAE), San José, Costa Rica, with assistance of Lourdes Vargas-Fallas and Javier Guevara-Sequeira.

**Figures 61–64.**
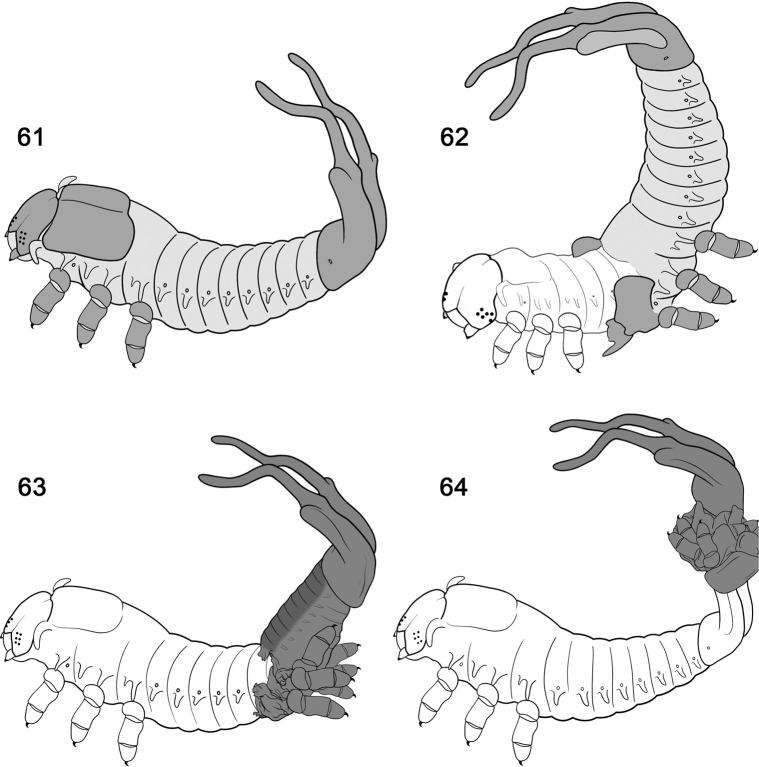
Timing of moulting process and exuviae retention in *Calyptocephalaattentuata*. **61** at 17 seconds. Instar I larva lacks the shield **62** at 7 mins, instar II exiting from instar I exuvia **63** at 8 mins, the old head capsule is folded caudad, the instar II pulls forward, pushing the exuvia posteriad **64** at 13 mins, instar II larva with exuvia of instar I on urogomphi. Other instars with additional exuviae (drawn by L. Schletzbaum; timing follows films (Yamamoto 2018, 2020).

**Figures 65–70.**
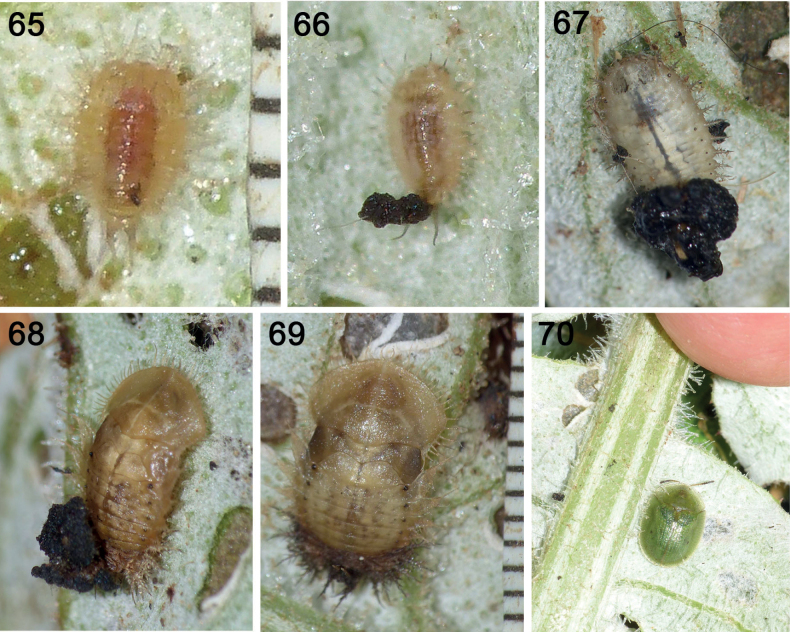
Life stages of *Cassidasphaerula* Boheman, 1853 (Cassidini) **65** instar I, neonate **66** instar II, with faeces on caudal processes **67** mature larva with faeces + exuviae shield **68** pupa with entire larval shield (faeces + exuviae) **69** pupa with shield comprised of only 5^th^ instar exuvia **70** adult (photographs: S. Adam, September 2021).

**Figures 71–76.**
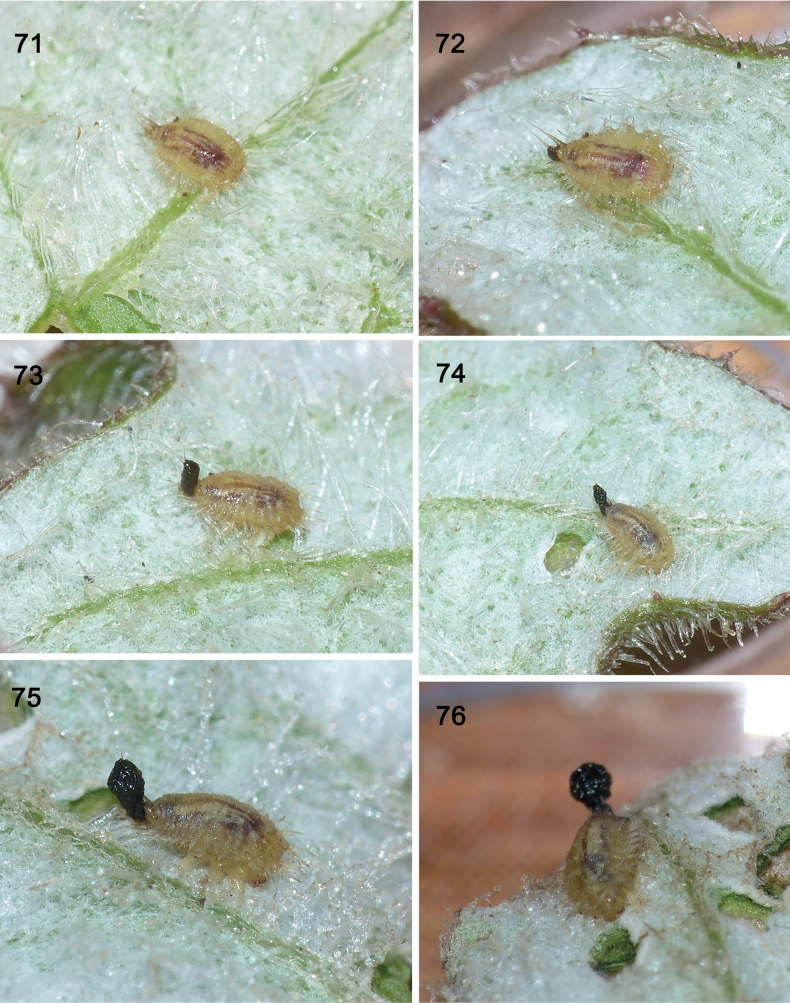
Re-construction of faeces on exuvio-faecal shield in Experiment 1, starting with instar I larva (so no prior exuvia), *Cassidasphaerula* Boheman, 1853 (Cassidini; photos: S. Adam, September 2021) **71** instar 1 (~ 2 mm long) at time 0 when faecal shield is removed, exposing urogomphi **72** larva at two hours, small faecal blob at anus **73** larva at four hours, urogomphi encased in faeces **74** larva at six hours, urogomphi encased in faeces **75** larva at 23 hours, lateral view. **76** larva at 48 hours, dorso-ventral view (photographs: S. Adam, September 2021).

**Figures 77–82.**
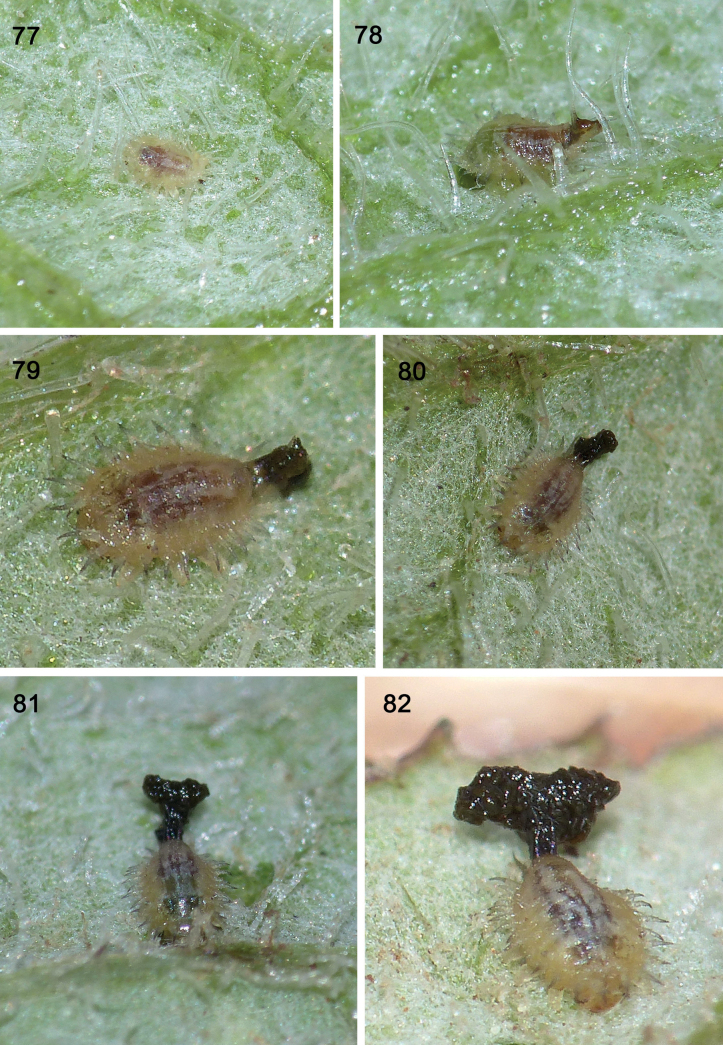
Re-construction of faeces on exuvio-faecal shield in Experiment 2 with *Cassidasphaerula* Boheman, 1853 (Cassidini) **77** instar I (~ 2 mm long) before shield construction **78** instar II at time 0 with faeces removed (scraped off) **79** after 2 hours, dorsal view **80** after four hours, dorsal view **81** after 23 hours **82** after 48 hours (photographs: S. Adam, September 2021).

**Figures 83–86.**
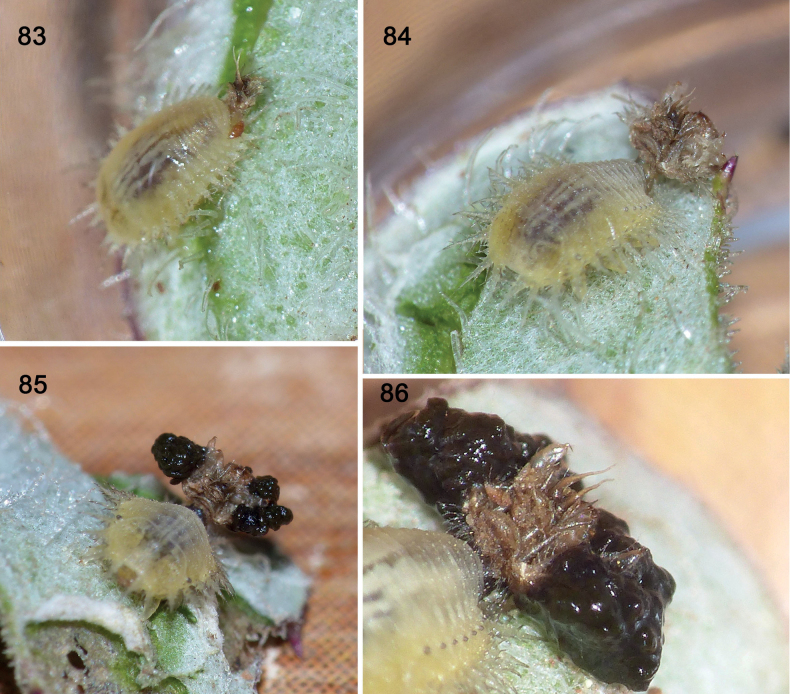
Faecal re-construction in experiment 3 with instar II larva, *Cassidasphaerula* Boheman, 1853 (Cassidini) **83** time 0 when faecal shield is removed, exposing instar I exuvia **84** larva at two hours, exuvia I still exposed **85** larva at four hours, faeces attached to lateral projections (scoli) of exuvia I **86** larva at six hours, exuvia I with a lot of faeces (photographs: S. Adam, September 2021).

### ﻿Photography and film

Various digital cameras were used for photography and filming KN used Nikon Coolpix E4500, Canon EOS 7D, Olympus STYLUS TG-4 Tough, and Sony α7S. The movie of *Calyptocephala* moulting was filmed with at 4K movie resolution using Sony’s digital camera “α7S” with Canon MP-E65mm F2.8 1–5× Macro Photo lens. SA used a Panasonic DMC-FZ200 camera plus a Raynox macroscopic lens M-150 and live individuals were observed with a Zeiss stereoscopic microscope plus a Dino-Lite eyepiece digital microscope/camera. CSC used a Basler camera attachment on a Nikon SMZ800 microscope. Photo editing was done in Paint.net or Photoshop. LS did the illustrations in Adobe Photoshop and Adobe Illustrator.

### ﻿Taxonomic names, morphological terms, phylogenetic characters

We follow the Cassidinae classification and taxonomic names of Staines (2015) and Borowiec and Świętojańska (2002–present). We follow Chaboo (2007) for morphological terms and phylogenetic character numbers discussed herein (see more discussion under Phylogeny below). Other group-taxon names for beetles follow Bouchard et al. (2011).

### ﻿Terminology

This section provides definitions of entomology and cassidine larvae terms that are used to describe the shield construction process. In addition to our illustrative plates, shields can be found in these other synthetic sources: Takizawa (1980), Chaboo (2007), Flinte et al. (2009), and Świętojańska (2009).

In holometabolous insects, larvae instars are demarcated by ecdysis events. Since the process of ecdysis lasts a few seconds (Hemimetabola juveniles are called nymphs), in practice, entomologists recognise the new instar starting when the previous instar’s exuvia separates from the epidermal cells of the new instar’s exoskeleton (a process called “*apolysis*”). The section aims to help readers understand the interactions between processes and parts involved in shield formation, described in the ‘Results’ section.

#### ﻿Exuviae

We use “exuvia” (singular) and “exuviae” (plural) for the exoskeletons (“skins”) shed at ecdysis following Snodgrass (1935) and Chapman (1982: 519). Entomologists have co-opted the Latin terms that translates as “things stripped off” (Latin is Simple 2023). Schuh (1989) recognises “exuviae” only. Exuvium is linguistically incorrect and hardly used. “Pharate” is used to describe when the exuvia is retained and encloses the teneral insect (Chapman 1982: 518); in tortoise beetles, the exuvia is retained without enclosing the emerging larvae, so the latter is not pharate. In *Aproida* Pascoe (tribe Aproidini), the pupa is suspended from the larval exuvia (Monteith 1970), probably by everted forgut cuticle lining as in some other beetles (Frania 2011); this is unlike the exuvial retention of tortoise beetles. We describe some shields below as exuvia-only (single exoskeleton of Instar II larvae and pupae?) or exuviae-only (with more than one exoskeleton).

#### ﻿Caudal process

‘Urogomphus’ (singular) and ‘urogomphi’ (plural) are used widely in insects, referring to the paired spine-like dorsal projections originating from the 9^th^ abdominal tergite of many larvae (Duporte 1977; Schuh 1989). They are not homologues of cerci, projections of the 11^th^ abdominal segment, nor are they universally homologous across Insecta. Within Chrysomelidae, the ten tribes of tortoise beetles (= the crown clade) in the Cassidinae share a character of larvae having paired projections (a few species secondarily exhibit a single process, Chaboo 2007: char. 11). Plesiomorphic ‘hispine’ larvae lack these dorso-caudal processes but some mining and cryptic feeders have their 9^th^ abdominal tergite modified, heavily sclerotised and concave, into a “urogomphal plate” (Maulik 1931; Chaboo 2007). The tortoise beetle processes are also not morphologically homologous with such processes in juveniles of other chrysomelid subfamilies (e.g., urogomphi in Chrysomelinae larvae *sensu*Reid 1992a, b), other beetle families, or other insects. In the chrysomelid literature, the cassidine caudal processes have been called many terms: posterior spikes (Kershaw and Muir 1907), anal furca (Buzzi and Miyazaki 1992), supra-anal furca (Heron 2007), and supra-anal processes (Borowiec and Świętojańska 2014). Some labelled figures of Cassidine structures are in Chaboo (2007: fig. 18F of larva and 19C of pupa) and Adam et al. (2022: figs 21–25). We use the term caudal processes here for Cassidinae but indicate “(= urogomphi)” in discussions below to remind readers who may be more familiar with that term.

#### ﻿Scolus, scoli

We follow the Torre-Bueno Glossary of Entomology (Schuh 1989) using both singular and plural terms for lateral projections from the thorax and abdomen of the larval and pupal body. Scoli are not homologous with tergal-originating caudal processes (= urogomphi). Cassidinae larvae and pupae may have scoli on the pronotum, metathorax and abdomen; these are unbranched and can be simple, spinose or have short setae.

**Anus** (Figs 45–50). Tortoise beetle larvae have a unique anus, sub-terminally-opening, muscular, extensible, and highly manoeuvrable unlike other chrysomelid larvae, which have a simple pore-like anus. The telescopic anus of Cassidinae likely represents the plesiomorphic abdominal segments X–XI. The anus is moved by peristaltic movements (Gómez et al. 1999).

**Shield** (Figs 19–26). This is attached to the caudal processes and held over the cassidine larval and pupal body, sometimes reaching over the pronotum. Annex, parasol, shield, and umbrella (Jolivet and Verma 2002) have been used to describe the structure. Tortoise beetle shields have been called other names: larval clothing (Muir and Sharp 1904), ‘kotanhang’” (= faecal appendage; Fiebrig 1910), faecal mask (Engel 1935), faecal shield (Eisner et al. 1967), faecal pad (Hawkeswood 1982), and exuvio-faecal annex (Buzzi 1988). Buzzi’s (1988) term is precise about materials (i.e., exuvio-faecal) and does not imply function (i.e., annex is neutral compared to shield). “Annex” is probably the best term, however at this time, shield has become so widely used in the literature and concurs with the experimental work demonstrating its functions, thus we will retain this term.

#### ﻿Faeces, frass, fecula

Many terms for insect excrement appear in the literature: excrement (Hislop 1872; Scudder 1891; Blatchley 1924; Muir and Sharp 1904; Flinte and Valverde de Macédo 2004), faeces (Snodgrass 1935), ‘faeces, fecula and frass’ (Frost 1942), excreta (Wood 1966; LeSage 1982; Jolivet and Verma 2002), scat (Lécaillon 1896; Hinton 1981), and fecula (Gómez et al. 1999). In this context, the term “faeces” is used to refer to waste substances emerging at the anus (Snodgrass 1935; Chapman 1982; Schuh 1989), which should not be confused with other exudations (honey dew, spittle froth, glandular and salivary secretions, etc.). Terms like merdigery (Jones 1994) and psammophory (Bameul 1989) refer to faeces and sand, respectively.

### ﻿Experiments to unravel shield architecture

Chaboo’s (2007) subfamily phylogenetic study of Cassidinae determined that the exuvio-faecal shield represents a unique morpho-behavioural complex supporting monophyly of tortoise beetles (10 tribes, ~ 2700 species). The majority of these species has exophagous larvae that retain the cast exuviae and apply their own faeces to build the distinct globular or pyramidal structure. This is held on their caudal processes and can be moved about. Within this crown clade, a few species do not retain a shield and we will discuss this pattern in our evolutionary discussion below.

Typically, a tortoise beetle female may deposit faecal pellets onto eggs or oothecae, but it is the instar 1 that initiates the shield with its faecal material. Instar II retains the exuvia of instar I on its own caudal processes and attaches its own faeces. For *Cass.sphaerula*, we dissected shields to understand how it is fitted and held together.

*Calyptocephalaattenuata* (Spaeth, 1919) (Figs 51–58). Observations and imaging were made over a 2-yr period by KN; specimens collected by KN were studied by CSC by dissection and imaging to determine how the shields are held together. The moulting process was filmed in Costa Rica for some Japanese television nature documentaries (Yamamoto 2018, 2020), assisted by KN; KN also photographed published nature notes (Nishida 2014, 2015; Nishida et al. 2020). We describe the moulting process and shield architecture under ‘Results’. The moulting process exhibits active and quieter periods; to ease description, we use 'phases' and timing to describe the sequence filmed.

*Cassidasphaerula* (Figs 65–89). Given access to a large population, we were able to access many live specimens for various manipulations indoor to document the construction, enlargement, and transfer of the exuvio-faecal shield from one instar to the next, then to the pupa. We observed multiple larvae of various instars indoors, maintained in plastic containers at ambient temperatures and light and supplied daily with fresh host leaves. We followed these larvae until the emergence of adults. We studied the effects of shield removal in three experiments as follows:

**Figures 87–89.**
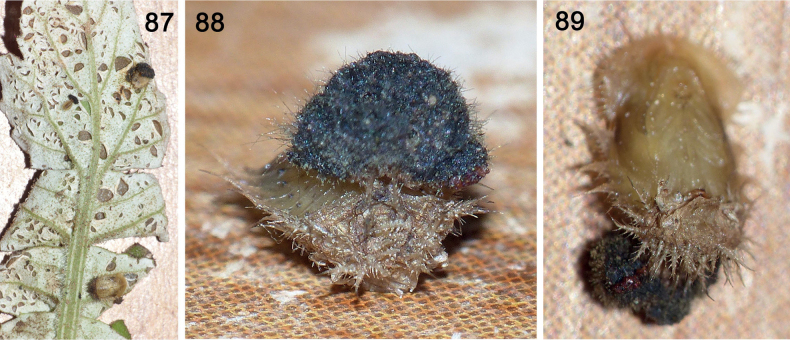
Shield of pupa of *Cassidasphaerula* Boheman, 1853 (Cassidini) **87** host leaf chewed by beetles, with one larva and two pupae (dorsal views; upper one with exuvio-faecal shield; lower one with exuviae-only shield) **88** posterior view showing exuvial-faecal shield (of instars I–IV) attached to caudal processes of instar V exuviae **89** ventral view showing complete instar V exuvia and exuvio-faecal shield (photographs: S. Adam, September 2021).

1. Experiments 1–2: remove the shield entirely, sliding the structure off the caudal processes and leaving the live larva naked.
2. Experiment 3: abrade only the faecal part of the shield, leaving the exuviae *in situ* on the caudal processes.

We photographed and filmed these individuals at 2-hr time (T) intervals to capture the initiation, expansion, and maintenance of the exuvio-faecal shield. We paid attention to larval movements and pupation. Based on these observations, dissections, and imagery, we describe the shield architecture, shield construction and reconstruction, and the moulting process under ‘Results’ below.

### ﻿Phylogeny relations

Faecal constructions are considered here at two levels, first in Chrysomelidae and second in Cassidinae. For Chrysomelidae (Figs 16–18), we only present the broad pattern of faecal constructions and their possible role in sub-familial diversification, so we simplify the original sampled taxa to subfamily names to show the overall topology of recent major analyses (Reid 1995, 2000; Gómez-Zurita et al. 2007, 2008; Nie et al. 2020). We do not discuss the underlying evidence and premises supporting these topologies.

For Cassidinae, subfamilial monophyly is well-supported in hypotheses of chrysomelid evolutionary relationships (Farrell 1998; Reid 1995, 2000; Gómez-Zurita et al. 2007, 2008; Hunt et al. 2007; Haddad and McKenna 2016; Song et al. 2017; Nie et al. 2020). The internal relations are not fully settled. Cassidinae were historically treated as two subfamilies, Hispinae (“hispines”) and Cassidinae (“tortoise beetles”), but are now recognised as a single subfamily, Cassidinae sensu lato, based on life history, morphological, and molecular evidence (Borowiec 1995; Hsiao and Windsor 1999; Chaboo 2007); other phylogenetic studies target subsets of tribes. Two online catalogues are available for “hispines” (plesiomorphic Cassidinae, 3,371 species in 24 tribes; Staines 2015) and for “tortoise beetles” (2,948 species in 12 tribes; Borowiec and Świętojańska 2002–present). Opinions differ about the status of certain tribes, arising largely from lack of natural history data, and are reflected in the catalogues (Staines 2015; Borowiec and Świętojańska 2002–present) and in higher-level phylogenies. For example, the catalogues overlap regarding Imatidiini and Spilophorini. These catalogues are still valuable and allow us to extract information on faecal-building behaviours from the documented life cycles. Chaboo and Engel (2009) examined the phylogenetic positions of two crucial fossils, *Denaeaspischelonopsis*Chaboo and Engel 2008 (tribe Imatidiini) and *Eosacanthadelocranioides*Chaboo and Engel 2008 (tribe Notosacanthini) at the transition zone between basal Cassidinae (“hispiforms”) and tortoise beetles (derived Cassidinae or Cassidinae sensu stricto) so these topologies are also pertinent to discussing the origins and timing of the shield-constructing behaviour.

## ﻿Results

We report on four tortoise beetle species from three tribes: Cassidini, Mesomphaliini, and Spilophorini. We outline the basic life cycle of tortoise beetles with two models, *S.cucullata* and Cassidini undet. sp. 4, and introduce special terminology and morphology used for tortoise beetle shields. Then we describe shield architecture, shield retention, and shield construction and reconstruction in *Cal.attenuata* and *Cass.sphaerula*, based on field observations and laboratory manipulations and dissections. We pay particular attention to the caudal processes and the telescopic anus in the two latter species to understand their roles.

### ﻿Natural history of *Stolascucullata* (Tribe Mesomphaliini) (Figs 41–44)

This species serves to outline the general life cycle of tortoise beetles and to explain special terms and definitions in Cassidinae. The female (Fig. 41) was captured and provided with a dry twig on which she deposited three eggs (Fig. 42). ***Egg.***Cassidinae eggs may be solitary or grouped, and some are even guarded by mothers (i.e., subsocial); in *S.cucullata*, the female oviposits a group but each egg is separated. Cassidine eggs may be covered with plant debris, oothecal membranes, or faecal depositions; in *S.cucullata*, the eggs are naked. They are initially white, then turn grey within a few minutes, then reddish brown with a black apical disc (Fig. 42). Egg size (n = 2: 2.4 mm long; 1.0 mm wide. ***Larvae*** (Fig. 44). The neonate larvae have a yellow body with yellowish cream scoli and are densely setose. They wandered away after hours/days, living a solitary life which contrasts with many tortoise beetles that maintain a gregarious group that can additionally be guarded by the mother (subsociality; Chaboo et al. 2014). ***Comments*.** The host plant, *Neomirandia*, has 56 known species and may host other *Stolas* species; its interesting chemistry (Tamayo-Castillo et al. 1989) is suggestive of a possible role in the beetle’s biology and its exuvio-faecal shield.

**Shield construction behaviour.** The natal larva (Fig. 44) has many scoli and paired caudal processes, all with long setation. As the larva feeds, faeces accumulate on these paired caudal processes and, it appears, are held additionally by the long hairs. We have not yet observed the other life stages of this species, but we note how the shield is initiated in Instar I.

### ﻿Natural history of Cassidini undet. sp. 4 (Tribe Cassidini) (Figs 45–50)

These larvae build a wide fan-like shield. ***Shield construction behaviour*** (Figs 45–50). At each moult, the exuvia is shed, from the head to the hind end, but is not cast off. Instead, the exuvia remains attached to the caudal processes. Faeces are added all over, enlarging the shield structure which becomes dry and black-brown in colour. We observed the long, telescopic anus extend and deposit faeces; the anus is highly manoeuvrable and can extend nearly 2/3 of the body length (note different positions of the anus in Figs 45–50). The shield becomes a large triangular structure with the exuviae stacked internally but not apparent externally, being so daubed over with thick faeces. ***Materials*.** The instar I initiates the faeces-only shield but later instars have a shield of all larval exuviae and faeces. This is inherited by the pupa (Figs 49, 50); note the fungal hyphae growing upon the shield. ***Associated morphology*.** The extensible anus builds the shield, placing wet faeces on the caudal process (instar 1) or on the exuviae + faeces of older instars. In older instars, the chaetotaxy is much smaller, raising a question if long chaetotaxy on the instar I caudal processes help hold on to moist faeces, until a hardened structure forms; older instars do not have such long chaetotaxy. The caudal processes in both larva and pupa provide the scaffold of construction (internally, the exuviae become inter-nested at their caudal processes, giving stability). In the larva, caudal processes also rotate the shield vertically, forward and lowered onto the dorsum, backward and extending flat behind the body, and side to side. This raises a question of stability of the larva’s body while moving such a relatively large structure; certainly, the feet must be firmly anchored, temporarily glued perhaps, on the leaf and stem substrate. The two caudal processes move but we do not know if each process can move independently of the other. In the solitary pupa (Figs 49, 50), we noted shields held in different positions, directly on the dorsum (Fig. 49) or backwards (Fig. 50). The pupa’s abdomen is firmly glued and anchored to the leaf substrate.

### ﻿Natural history of *Calyptocephalaattenuata* (Spaeth, 1919) (Spilophorini) (Figs 51–56)

Illustrated natural history notes have been reported (Nishida 2014, 2015; Yamamoto 2018, 2020; Nishida et al. 2020). Shield construction behaviour: The larvae retain a shield comprised solely of exuviae of previous instars on the paired caudal processes (“urogomphi”) (Figs 51–54). The mature larva carries five exuviae (Figs 51, 52), thus indicating that larva as Instar VI and this is an atypical life cycle for Cassidinae (Chaboo 2007).

The process of shield-building in *Cal.attenuata* begins at the end of Instar I. We describe this process, based on field data and photographs of KN (Nishida 2014, 2015; Nishida et al. 2020), including his assistance on staging the beetle to film the behaviour for two nature documentaries (Yamamoto 2018, 2020; we indicate time (T) in minutes and seconds below based on the film, but readers must access film).

1. **Phase 1** (Fig. 61). Larva, instar I (~ 4.1 mm long), naked, lacking a shield. The larva becomes quiescent as it prepares to moult (T 0–1 min). The six legs are firmly anchored on the leaf and the claw tips appear to be a little embedded on the leaf surface. With a few large inspirations, air fills the gap between the old instar I exuvia and the new instar II; the former seems to lift away. Then the old thoracic nota split medially (T 1 min 35 secs). The abdomen and caudal process move slowly and gently forward and back. The larva inspires air again, inflates a little, and the new prothorax pushes out of the old skin (T 2 mins 5 secs), further widening the breach along the notum. The head capsule splits along the epicranial suture (T 2 mins 28 secs); the new prothorax pushes out further (T 2 mins 40 secs), freeing the lateral scoli (T 2 mins 38 secs), and pulling the head out (Time 3 mins 5 secs). The head and thorax are lifted and freed of the exuvia I, then the new legs are lifted free of their old exoskeleton (T 3 mins 16 secs - 3 mins 25 secs); the instar II abdomen is still encased in instar I abdominal exoskeleton that has not yet split open (Fig. 62).
2. There is a pause as the head, thorax and legs are lifted vertically, with only slight movements of new legs. The instar II integument is white; yellow haemolymph is apparent internally at the coxal bases. The six pairs of stemmata are black.
3. **Phase 2** (T 6 mins 30 secs - 6 mins 44 secs). Exuvia II legs drop to the surface, then position on the leaf rib and surface, perhaps anchoring claws into the substrate. The entire body heaves a little, gently, then faster, pulling the instar II abdomen free of the Instar I exuvia. The instar I legs lift free of the substrate. Instar II does not walk forward, but pro- and meso-legs stay fixed on the vein as at the start of Phase II. The body is now lifted and rotated, in 360°, extending the abdomen which pushes the anterior section of the old exoskeleton further posteriad (T 7 mins 10 secs). The larva heaves the body anteriad and posteriad, pushing the instar I exuvia backwards (T 6 mins 53 secs). The metaleg positions and re-positions during this phase. The abdomen is held close to the substrate allowing the old head capsule to be dragged against the substrate and pushed further posteriad. At T 7 mins 36 secs, abdominal segments I–II become liberated of the old exoskeleton; shortly after, most of the larval abdomen is extracted from the old exuvia (Fig. 63). By T 9 mins 31 secs (Fig. 64), the old exuvia has been pushed to the posterior half of the new caudal processes (In the sped-up film, the process looks violent). The entire process takes about 17 mins in real time.
4. **Phase 3.** T 11 mins, it appears that abdominal sternites I and II may anchor to the substrate. The anus appears protuberant. The larva sits for another 6 mins before it slightly repositions all its legs. The exuvia of instar I is now positioned on the posterior half of the new caudal processes, with the body folded over and its caudal processes free. The abdominal section of the exuvia is oriented anteriad; the legs, thorax and head sections are folded over and oriented posteriad. Only the posterior half of instar II’s caudal processes are inserted into exuvia I, holding it together.
5. **Phase 4.** T 14 mins 3 secs, the instar II larvae changes position and we gain a posterior view of its abdomen and caudal processes. The curvature and width of the new caudal processes retains the exuvia I firmly, with some tension.

1. ***Instars II* – *V* .** These instars were not observed, but ecdysis at the end of each instar probably follows a similar process as above, with the preceding shield retained on the posterior section of the caudal processes.
2. ***Pupa* .** The mature larva attaches the abdomen to the leaf and undergoes pupation. Of the pupae collected, all retained a shield; some shields comprised of two or three older exuviae, but not the younger exuviae that would be most apical in the stacked structure. These shields extended only up to the pronotum, so it is possible that the younger exuviae fell off during ecdysis or were subsequently abraded. Figs 57 and 58 show a pupa with five exuviae, suggesting that exuvia I is detached (these pupae could have six larval exuviae); thus, the pupa inherits shields with varying numbers of exuviae. Figs 57–58 also show the teneral adult partly exiting this pupal exoskeleton.

#### ﻿Materials

The larval shield of *Cal.attenuata* is comprised only of exuviae; there are no faecal deposits, secretions, nor plant materials.

#### ﻿Morphology

Roles of caudal processes in larva and in pupa. These are critical to retaining the shield on the body and to connecting all the previous exuviae together in a single structure. The posterior sections of each caudal process are entirely enclosed within the previous exuvia. ***Repair*.** It seems obvious that the larvae have no way to repair these exuviae-only shields; if one or more exuviae are removed, the larva must wait until the next moult to add a new exuvia. The movements of the abdomen and caudal processes are responsible for moving the shield in various directions, forwards, laterally and backwards, including above the head. The pupae lack the processes; instead, the final larval exuvia is wrapped around the pupa’s caudal region and retains the larval exuvial shield. Some shields (Fig. 59) in our unidentified Ecuadorean Spilophorini have leaf fragments attached; these are possibly accidental.

### ﻿Natural history of *Cassidasphaerula* (Cassidini) (Figs 65–89)

*Cassida* Linnaeus, 1758 comprises 484 species (Borowiec and Świętojańska 2002–present). Immatures have been described for 64 species and exuvio-faecal shields have been noted in most documented larvae to date (Świętojańska and Borowiec 2007: Table 1; Świętojańska et al. 2013). Natural history of *Cass.sphaerula* was reported by Adam et al. (2022) and we summarise in Figs 65–70. Females oviposit small clusters of eggs with oothecal membranes, there are five larval instars (Figs 65, 67), all solitary, and pupae are solitary (Figs 68, 69).

### ﻿Shield construction behavior

Soon after the natal larva (Fig. 65) begins feeding, it begins accumulating its faeces on its paired caudal processes (Fig. 66). At each moult, the cast exuvia is pushed to the base of the caudal processes. The shield becomes a rough triangular-shape, with dark brown-black faeces obscuring the lighter-brown exuviae slightly visible at the base (Fig. 68). The pupa retains the entire larval shield of exuviae + faeces (Fig. 68) or retains only the 5^th^ larval exuvia (Fig. 69). The faeces are dense, at different times appearing wet, moist, or desiccated.

#### ﻿Incorporation of exuviae into shield

At ecdysis, the old exuvia splits along the ecdysial line of the head and is peeled and pushed backwards, as the teneral instar pulls forward to free its legs. It fixes the legs to the leaf surface, then wriggles its abdomen forward to free itself of the old exuvia. In this way, the previous exuvia becomes positioned at the base of the caudal processes of the teneral larva, beneath the existing exuvio-shield structure. Since all the caudal processes are nested (all previous exuviae atop the living caudal processes of the current instar), the former exuvia becomes crumpled at the base of the existing shield. Soon this recently added exuvia becomes daubed with faeces, and so becomes indistinguishable within the entire shield structure (unless the latter is dissected). No exuviae are omitted from the central scaffold. Apart from the shield structure, excess faeces may be left on the leaf.

We address the question “Will larvae rebuild the shield” with several shield-removal experiments to observe responses of larvae. We present results of three experiments below.

#### ﻿Experiment 1, instar I (Figs 71–76)

T 0 mins, (Fig. 71): Shield removed completely, exposing the living paired caudal processes. T 2 hours, (Fig. 72): a small quantity of faeces accumulates on the anus. T 4 hours (Fig. 73): moist faecal material has accumulated on the urogomphi, covering it up to the apices. T 6 hours (Fig. 74): faecal material almost the same as at T 4 hours. T 23 hours (Fig. 75): The faecal shield is almost twice as large. T 48 hours (Fig. 76): The faecal shield is about three times larger than it was at T 2 hours.

#### ﻿Experiment 2, instar I (Figs 77–82)

Time 0 (Fig. 77): We removed the shield entirely, both exuvia I and faeces. T 2 hours (Fig. 78): a small amount of fresh faeces accrue on the caudal processes. T 4 hours (Fig. 79): more new faeces accumulate, obscuring the caudal processes. T 6 hours (Fig. 80): more new faeces accumulate. T 23 hours (Fig. 81): the faeces have grown into a small, dimensional inverted pyramid. T 48 hours (Fig. 82): the inverted pyramidal shield is larger, held together on the caudal processes. This shape seems unstable, that lateral sections should break off yet hold together.

#### ﻿Experiment 3, instar II (Figs 83–86)

T 0 mins (Fig. 83): we scraped away only the faeces to expose the Instar I exuvia still attached to the caudal processes. T 23 hours (Fig. 85): faeces have been applied to the sides of the old exuvia, so the overall shield width is almost as wide as the larva. T 48 hours (Fig. 86): More faeces have been applied to the lateral margins, so the shield is now a little wider than the larva. The old exuvia is in the centre, exposed, and the moist black faeces hang on to the sides.

The entire exuvio-faecal structure was gently eased off the living caudal processes using forceps and these intact larvae continued feeding. In each case, the larva soon produced a faeces-only shield, small at 2 hours after removal, then bigger and bigger at hours 4 and hours 6 after removal. By hours 23 and 48, 1–2 days after the earlier removal, the new shield was larger and club-shaped. In the three experiments of shield manipulation, the timing, and responses to reconstruct a new shield were similar. The experimental larvae of *Cass.sphaerula* moulted normally and retained the exuvia into the inherited shield.

The larva can rotate the shield in a circular plane over the body, forward up to the mesothorax, and backward almost 180°, and in a horizontal plane with the body (Suppl. material 1). Films of the acrobatic movements of the larva’s extensible anus reveal that it applies faeces to the existing shield and, also periodically exudes large, mostly clear, droplets that are applied to and absorbed into the shield (Suppl. material 2). We found no trichomes in dissected shields even though we observed consumption of trichomes in *Cass.sphaerula* (Adam et al. 2022).

#### ﻿Shield retention in pupae (Figs 87–89)

In *Cass.sphaerula*, we observed pupae can have either an exuvia-only shield (Figs 69, 87) or the entire inherited exuvio-faecal shield structure of the earlier larvae (Figs 68, 88, 89). The faeces of the latter are dry since new faeces are not being applied. We found several discarded exuvio-faecal shields next to pupae. Given the observation of the moulting process (from 6^th^ instar to pupa) in *Calyptocephala* (described above), we infer that pupation is similar, with splitting of the ecdysial sutures on the cranium and thorax of the 5^th^ instar split and the pupa pulls forward and out. In the larval moults, the new legs and the old legs serve to anchor the emerging larva at different times in the process.

## ﻿Discussion

Faecal-based constructions and faecal debris-carrying are widespread behaviours in Chrysomelidae. Chrysomelid faecal-based constructions have been studied in terms of ecological function (Olmstead and Denno 1992; Gómez 1997, 2004; Morton 1997; Vencl and Morton 1998a, b, 1999; Morton and Vencl 1998; Gómez et al. 1999; Müller and Hilker 1999, 2001a, b, 2003, 2004; Vencl et al. 1999, 2005, 2011; Nogueira-de-Sá and Trigo 2002, 2005; Bacher and Luder 2005; Bottcher et al. 2009; Huang et al. 2022). Until now, this faecal building behaviour has been studied in only one chrysomelid, *Neochlamisus* by Brown and Funk (2005).

### ﻿Materials in cassidine shields

The macro-materials in shields of our observed species comprised exuviae only or faeces + exuviae. These two materials are side effects of metabolism and moulting respectively. Additional analyses may identify other possible components (Table 1) and their functions. The construction processes we documented allow us to now analyse how the two primary materials originate, are manipulated into the construction, and are held to the body. We briefly discuss evolutionary insights as we compare these aspects with other Cassidinae and other Chrysomelidae.

**Table 1.** Architects and materials used for faecal-based shields in subfamilies of Chrysomelidae: Cassidinae (Chaboo 2007; Świętojańska 2009), Criocerinae (Vencl et al. 2004), Cryptocephalinae and Lamprosomatinae (Chaboo et al. 2016), and Galerucinae (Prathapan and Chaboo 2011). Comparison of life stages, materials of larval/pupal faecal-based cases and shields, and larval body parts for construction. Key: + = present; – = absent; ? = unknown.

Feature		Cassidinae: 10 tribes, tortoise beetles	Chrysomelinae: *Phola* sp.^8^	Criocerinae	Galerucinae: Alticini: *Blepharida*-group	“Camptosomata”	
						Cryptocephalinae	Lamprosomatinae
**Stage**	Mother	–	?	–	+	+	+
	Egg	+/–	?	–	+/–	+	+
	Larvae	+/–	+	+	+	+	+
	Pupae	+/–	?	–	?	+	+
**Larval/pupal material**	**Endogenous**						
	Faeces	+/–	+	+	+	+	+
	Exuviae	+/–	?	+^1^/–	–	–	–
	Chemicals	+/–	?	+/–	+/–	?	?
	Waxes	?	?	?	?	?	?
	Saliva	?	?	?	?	?	?
	Regurgitates	?	?	?	?	?	?
	**Exogenous**						
	Soil	–	?	–	–	+/–	+/–
	Debris	–	?	+	–	+/–	+/–
	Trichomes	–	?	–	–	+/–	+^9^/–
	Leaf fragments, fresh	_	_	_	_	+/–	?
	Leaf fragments, decomposed	_	_	_	_	+/–	?
	Bark, twigs	–	–	–	–	+^5^/–	?
	Chemicals	+/–	?	+/–	+/–	?	?
	Fungi	+^7^/–	?	–	–	+/–	?
	Micro-organisms	?	?	?	?	?	?
**Morphology**	Abdomen	+	?	–	–	+	+
	Caudal Process	+	?	–	–	–	–
	Setation	?	?	?	?	?	?
	Anus	+	?	+	+	+	+

^1^*Lemajacobiana* Linell includes exuviae in faecal coat (Kaufmann 1967). ^2^Waxes were reported in *Saxinissaucia* LeConte, 1857 (Spruyt 1925) and in *Fulcidaxbacca* (Bokerman 1964). ^3, 4^*Neochlamisus* use saliva mixed with faeces in cases (Briggs 1905; Brown and Funk 2005). ^5^*Fulcidaxcuprea* (Klug, 1824) females integrate bark in egg-cases (Bokerman 1964). ^6^*Podontialutea* (Olivier, 1790) include exuviae in faecal coat (Takizawa 1978). ^7^Fungi was found in larval shields of Laccoptera (Sindia) sulcata (Olivier, 1808) (Rane and Ghate 2005) and *Cyrtonotasericinus* (Erichson, 1847) (Cedeño-Loja and Chaboo 2020); mycelia can be seen in other shields (e.g., *Canistra*, Flinte et al. 2009). ^8^*Phola* Weise, 1890 (Chrysomelinae) reported by Chen (1964, 1985). ^9^Described in Chaboo et al. (2008). Questions about Lamprosomatine cases arise due to their close relationship to Cryptocephaline cases that suggests possibly similar materials and constructions.

### ﻿Building stages in Cassidinae?

Larvae are the builders in our four studied species and building begins in two possible ways: 1) during instar I when faeces are deposited and held on the caudal processes as the larva feeds (in *S.cucullata*, Cassidini undet. sp. 4, *Cass.sphaerula*) or, 2) in the transition moult from instar I to instar II when the cast exoskeleton is retained on the caudal processes (*Cal.attenuata*). Cassidinae pupae in tortoise beetle tribes are not active builders; they receive their shields as an inheritance from the final larval instar and their shield is either the entire exuvio-faecal shield or only the final instar exuvia. Given the life cycle of Spilophorini, the final instar could be the 5^th^ or 6^th^ for tortoise beetles. For pupation, the pre-pupa anchors itself by gluing the abdomen to the host surface. Then the larval exoskeleton splits along the head and thoracic midlines and the pupa pushes out as the larval exuvia is propelled caudad. The shield is retained passively, attached on the pupa’s own caudal processes. This pupal inheritance recalls that of camptosomate chrysomelids where the final instar seals the larval faecal case to the substrate and so provides a pupation chamber (Chaboo et al. 2008).

### ﻿Cassidinae shield architectures

The common pattern is the exuvio-faecal shield built by larvae, retained in all instars, and which may be inherited by pupae. The faeces are variable in moisture, from desiccated (Figs 19, 22, 23, 25, 26) to wet (e.g., *Plagiometrionaflavescens* (Boheman, 1855): Flinte et al. 2009); this is certainly tied to the excretion of water, retention by Malpighian tubules, and rectal resorption. Dried faeces can be in long strands; these strands are arranged in a circular heap in Hemisphaerotini (Fig. 19; Chaboo and Nguyen 2004) or are held as vertical strands (Figs 22, 23). Within the tribe Ischyrosonychini, larval shields are varied: desiccated stacked exuvio-faecal shields (e.g., *Cistudinellaobducta* (Boheman, 1894) (Fiebrig 1910; Buzzi 1988), wet faeces (e.g., *Physonotaunipunctata* (Say, 1823); Keefover-Ring 2013, 2015), or older larvae that lack shields altogether (e.g., some *Physonota* Boheman, 1854). Larvae of *Eurypepla* Boheman, 1854 have a unique tapered body that is curved verticad, allowing wet faeces to slide down and coat the body (Chaboo 2004).

Architectural elements of cassidine faecal structures may be diagnostic for species-, genus- or tribal-level diagnoses. Shield architecture is determined by how exuviae are compressed and how faeces are arranged (long vertical strands, a dense clump, or a fan). Basket-like shields are diagnostic of Hemisphaerotini (Fig. 19; Eisner et al. 1967; Beshear 1969; Chaboo and Nguyen 2004) and appear to have some limited mobility, particularly in younger stages (note its position in Fig. 19). As the larval shield enlarges, it becomes less mobile, suggesting that this shield is relatively heavy and/or the caudal processes may not be as freely mobile. Although exuviae are retained in Hemisphaerotini, these are so compressed that only torn remnants remain at the base of the caudal processes, and it seems impossible to determine how many distinct exuviae are held. This shield architecture has been demonstrated to be protective (Eisner and Eisner 2000a).

We propose here that the particular exuviae-only shield architecture described herein is diagnostic for Spilophorini (Figs 51–54, 57, 59, 60). Life cycles of two species of *Calyptocephala* Chevrolat, 1836 (Buzzi and Miyazaki 1992; Córdova-Ballona and Sánchez-Soto 2008) on palm hosts reveal larvae with paired caudal processes and exuviae-only shields. Maulik (1932) described the larva of an *Oediopalpa* Baly, 1858 species with paired caudal processes and an exuvial shield; Chaboo (2007: 184) examined larvae in this genus and noted the unique pattern of exuviae compression. Hsiao and Windsor (1999) determined *Oediopalpa* as most closely related to *Calyptocephala* and *Spilophora*Boheman 1850 and Staines (2002) re-classified it in Spilophorini. Sekerka et al. (2014) reported an Orchidaceae host, the larval form, and exuviae-only shields for one species of *Cladispa* Baly, 1858 (Spilophorini). Monophyly of Spilophorini has been supported by adult characters (Chaboo 2007) and molecular data (Sekerka et al. 2014). The documented larvae exhibit exuviae-only materials arranged in a similar architecture, with a stable exuvial stack, a distinct spatial arrangement, and large partly exposed caudal processes. The exuviae are compressed and curved so the head capsule and the anus are exposed in posterior view. The shape of the caudal processes, like the yoke of a lyre, is unique in Cassidinae; the exposure (Fig. 57) of the large basal section of each process is also unique. These features altogether support monophyly of Spilophorini.

Other tortoise beetles exhibit exuviae-only shields (Figs 15, 21, 24) but the spatial arrangement of those exuviae and the underlying caudal process morphologies are unlike those in Spilophorini. In other documented species, exuviae are compressed differently, more closely at caudal processes, and head capsules are exposed in distinct ways (see examples: *Stolasimplexa* (Boheman, 1850), Flinte et al. 2009: pl. 18K; *Chiridopsisundecimnotata* (Boheman, 1855), Świętojańska 2009: fig. 128). As species and their constructions are documented, it may be possible to diagnose more groups based on more shield and process features.

Shields may be present or absent in pupae of tortoise beetles. We found that pupae of *Cass.sphaerula* retain different shields (the entire structure or only the final exuvia). Some pupae retain only the 5^th^ instar exuvia and their caudal processes are a dominant exposed feature (e.g., *Anacassis* Spaeth, 1913, Buzzi 1975; *Discomorpha* Chevrolat, 1836; Flowers and Chaboo 2015). In the Cassidini, pupal shields are known in species of *Charidotis* Boheman, 1854, *Drepanocassis* Spaeth, 1936, *Metriona* Weise, 1896, and *Syngambria* Spaeth, 1911 (Buzzi 1988). In some cassidines, the 5^th^ exuvia is retained by the pupa, encircling the terminal abdominal segments, e.g., *Anacassis* Spaeth, 1913 (Buzzi 1975, 1996). In *Eugenysacolumbiana* (Boheman, 1850) (Chaboo 2002), *Dorynotapugionota* (Germar, 1824) (Buzzi 1976), and *Chelymorpha* Chevrolat, 1836 (Buzzi 1998) this exuvia becomes part of the pupal attachment to the substrate. Shield removal is required to determine if this exuvia is wrapped around the base of the abdomen only or if it is attached to a pupal caudal process.

### ﻿What is the building equipment in Cassidinae?

We documented the anus moving freely over the posterior surface of the shield (Figs 45–48). We observed anal droplets excreted and quickly absorbed into the shield (Suppl. material 2). We also documented the application of fresh moist faecal deposits to the intact shield and, in our experiments, application to the exposed exuviae to rebuild the shield (Figs 71–86). Gómez (1997) reported the repair of damaged shields with precise deposits of faeces. Thus, the anus is the applicator for constructing and repairing the shield and appears to replenish the shield with moist droplets. Certainly, the cassidine anus has manipulative skill for these distinct roles (applying, building, repair, replenishment). Such replenishment may involve chemicals that sustain the shield’s chemo-barrier functioning. If pupal shields are not being replenished, this raises a question about their chemistry and functional effectiveness versus larval shields.

The musculated extensible anus of larvae is a second synapomorphy of the ten tortoise beetle tribes. Plesiomorphic Cassidinae larvae which do not exhibit shield-retaining behaviours have the typical posterior or ventrally opening simple anus pore and also lack caudal processes. As far as we know currently, no other chrysomelid larvae have an extensible anus. One question we have is the status of the anus in those Cassidinae with exuviae-only shields; we were unable to determine this in *Cal.attenuata*. Pinpointing the first appearance of the telescopic anus on phylogenetic topologies is one crucial element in the assembly of shield building traits.

Cassidinae larvae do not use their legs or mouthparts as building tools. Females may defecate on their eggs, but their genitalia lack rectal plates (as in Camptosomata: Erber 1968, 1969, 1988). In Camptosomata, the larva’s arrangement within its case positions the anus near the mouthparts and legs. Brown and Funk (2005) reported that faeces are mixed with a regurgitated yellow fluid and then applied to the margin of the case to continue building it or to repair holes, so the larva’s position with the mouth, anus and legs in proximity allows the faecal mixing and manipulation. Camptosomate larvae use their mouthparts to cut a longitudinal section which is then filled with faeces; this expands the girth of the case to accommodate the growing larva (Brown and Funk 2005; Chaboo et al. 2008). Calcetas et al. (2023) reports that *Podontia* larvae use legs and mouthparts to manipulate soil and faeces to build the pupation chamber.

### ﻿Building routines in Cassidinae

Cassidinae larvae use simple materials in simple building routines. Each shield has a distinct appearance due to the compression pattern of individual exuviae (Figs 15, 21, 24, 51, 52, 57, 59, 60) and due to the arrangements of faeces (strands, blobs, fan, bird nests, etc.). The shield enlarges at each transformation to the next instar as another exuvia is added basally to the mass. The extensible anus deposits faeces precisely on various parts of the exuviae to give the distinct appearance of shields. Faeces are extruded moist or wet, allowing attachment to the existing structure, before drying. Our simple experiments allowed us to understand the repair of the shields. If a portion of faeces is removed or broken off on one side of the structure, the anus can repair the faecal part to apply fresh faeces to recover a more balanced shield. Our study demonstrates that the shield-constructing behaviour is intrinsic and is probably not requiring any external activator to elicit the building response.

#### ﻿Role of caudal processes (= urogomphi)

Many animals that retain debris covers possess fastening structures, frequently specialised chaetotaxy (e.g., Weirauch 2006). We determined here several roles of the paired caudal processes— the anchorage or fastener for cast exuviae and faeces to the body, part of the shield materials, the crucial central scaffold by their inter-nesting, and movement of the shield. During instar I, faeces are applied directly to the caudal proceses; in *S.cucullata* dense chaetotaxy around the anal area may enhance faecal retention.

The exuvia is added to the shield with each moult, expanding the area for faecal attachment. In some species, exuviae alone make up the shield. The caudal processes become inter-nested from instar to instar, further strengthening the central scaffold of the exuvio-faecal shield and provide mobility, allowing it to be moved as needed to startle or hit an attacker or be the distasteful barrier. The caudal processes move the shield for a more active defence.

In pupae, we have no reports of cassidine pupae moving their shields, although there are reports of pupa jerking reflexively when disturbed (even in unison in gregarious pupae). It appears the entire pupal body jerks so pupal caudal processes may not be mobile.

#### ﻿Role of chaetotaxy

In one unidentified species and in *Cass.sphaerula* we observed that dense chaetotaxy in the caudal area of the neonate larva appears to aid initial faecal build-up. Specialised chaetotaxy may aid faecal retention in the faecal retaining chrysomelid clades. Specialised setae to hold on to debris have been described in unrelated beetles (Leschen and Carlton 1993; Leschen 1994; Yoshida and Leschen 2020), in other insects (e.g., Reduviidae, Weirauch 2006), and in other animals (e.g., spiders, Duncan et al. 2007; Gawryszewski 2014). In *Uraba* caterpillars (Fig. 9), it is a question how the old head capsules become stacked on the living caterpillar’s head, since the head capsule typically splits first during the moulting process, then becomes distorted as it is pushed posteriad, and the larva propels forward to exit its old exoskeleton. We suspect that specialised chaetotaxy on the caudal processes of tortoise beetle larvae and on the dorsum of larvae in Criocerinae and in the *Blepharida*-group may hold onto the faecal debris. Each debris-retaining animal has different strategies for attaching and retaining debris.

### ﻿Materials of coats, cases, and shields across Chrysomelidae (Table 1)

Chrysomelid constructions are composed mainly of endogenous faeces and, in Cassidinae, of exuviae. Documented exogenous materials are soil, fungi, leaf fragments (fresh, undigested, decayed), plant extracts, and trichomes (Table 1). We will not review here how exactly these materials may be mixed or intermingled with the other structural materials. In Cassidinae, fungal elements have been noted but not identified taxonomically (Figs 49, 50; Rane and Ghate 2005; Flinte et al. 2009; Cedeño-Loja and Chaboo 2020). Fig. 59 shows a larva with plant fragment on the exuvial shield but this may be accidental. It has been well-established that animal guts are rich with microbiota that can be passed to the next generation via the egg surface; Stammer (1935) established such transmission in Cassidinae. Faeces are also rich with microbiota (thus, Faecal Transplant technique); we can presume that the cassidine shield is harbouring microbiota that await discovery and study. The exuviae are a low-cost material that add substantial structural value to the shield (like straw added to dung) but we do not know yet their chemical contributions. All debris materials have pros and cons, depending on how they originate (time to produce or assemble) and their consequences (e.g., weight, odour, chemistry) so every chrysomelid material likely has a functional role simply because of the cost in carrying the weight and bulk of a structure; it is unlikely that unnecessary materials are selected. Most of these chrysomelid materials are actively manipulated, although it is possible that some (e.g., blown soil) may be passively integrated.

### ﻿Chrysomelid construction behaviours: ecological implications

Chrysomelid larval and pupal shields are hypothesised to serve multiple functions, including protection from extreme temperature (Réaumur 1737), humidity, precipitation and desiccation (Weise 1893), camouflage or mimicry (e.g., bird or caterpillar droppings: Briggs 1905; Blatchley 1910; Jenks 1940; Balsbaugh 1988; plant detritus: Lee and Morimoto 1991a, b), as a distasteful physical barrier deterring predators and parasitoids (Réaumur 1737; Eisner et al. 1967; Olmstead and Denno 1992; Olmstead 1994, 1996; Eisner and Eisner 2000a; Bacher and Luder 2005), or as chemical deterrents from exocrine glands of retained exuviae (Olmstead 1994). They can also be used as a mobile club to hit intruders or as a protective umbrella (CSC, pers. obs.). The term ‘shield’ implies passive protection, which may lower the body temperature or decrease wind shear (Olmstead and Denno 1992). The material and consistency (including cementing and chemistry) must ease accumulation and attachment. It appears that chrysomelid shields are generally resistant to rain as they do not absorb water and fall apart.

### ﻿Testing of function hypotheses

The hypothesis of a mechanical defence against predators has been tested experimentally and found to be supported (Eisner et al. 1967; Wallace 1970; Olmstead and Denno 1992; Eisner and Eisner 2000a; Schaffner and Müller 2001; Müller 2002). Blum’s (1994) hypothesis of defensive chemicals in shields has led to some analytical studies, usually of single chrysomelid species, aimed at comparing compounds in the faecal shields and the host plants (Mummery and Valadon 1974; Morton and Vencl 1998; Gómez et al. 1999; Vencl et al. 1999; Aregullín and Rodríguez 2003; Bacher and Luder 2005; [Bibr B221][Bibr B231]; Vencl et al. 2005, 2009, 2011; Bottcher et al. 2009; Keefover-Ring 2013, 2015; Vencl and Srygley 2013). Maybe a chemical barrier is achieved by integrating plant tissues and trichomes or by applying secretions (plant-sequestered or de novo chemicals) that volatilise around the animal, maybe creating a small chemosphere. Exuvial glands may have residual chemicals that may disguise the wearer or deter enemies.

In testing Ehrlich and Raven’s (1964) “escape and radiate” hypothesis, Vencl et al. (2011) compared differential functioning in defence of shields with/without faeces, larval solitary/gregarious living, and maternal care and deduced a sequence of trait accumulation correlated with enhanced defences and, likely, species diversification. Such creative experiments can assess the contribution of each trait within the defense array.

Others have determined the shields to have mixed effects, deterring some predators yet attracting others (Müller and Hilker 1999; Bacher and Luder 2005; Huang et al. 2022). Certainly, faeces can have chemical signatures that attract enemies (Van Leerdam et al. 1985; Agelopoulus et al. 1995).

Chemical deterrents in exocrine glands of retained exuviae (Hinton 1951; Olmstead 1994) have not been investigated. Furthermore, the traits accumulated in the defence arsenal must now include the morphological features that accompany the structures; for example, caudal processes enhance shield mobility in tortoise beetles and may enhance defence success. Our research here highlights the morphological features used by tortoise beetle larvae within their arsenal of weapons.

### ﻿Construction behaviours: evolutionary implications

The primary hypotheses proposed to explain chrysomelid hyperdiversity have been their ancient age (Farrell et al. 1992), herbivory and the rise of angiosperms (Farrell 1998), adaptive radiation with plants (Gómez-Zurita et al. 2007), and chemical adaptation to plants (Farrell et al. 1992). However, the great unevenness in subfamilial diversity begs for additional explanations. Transitions to new habitats within Chrysomelidae (e.g., aquatic, seeds, subterranean, mosses) and to the jumping escape mechanism (in ~8000 flea-beetle species, Begossi and Benson 1988; Furth 1988) await finer-scale study of correlated adaptations in morphology, physiology, and behaviour. Behaviours such as cycloalexy (larval defence formations; Jolivet 1988b), sound production (Schmitt 1994), myrmecophily (Agrain et al. 2015), and subsociality with maternal care (Chaboo et al. 2014) probably impact speciation in more restricted clades of Chrysomelidae. On the available phylogenetic hypotheses of Chrysomelidae (Figs 16–18), faecal armours appear as independent macroevolutionary events in speciose clades (part of Cassidinae; Cryptocephalinae; Lamprosomatinae) and in minor lineages (*Blepharida*-group within Galerucinae; Criocerinae; *Phola* within Chrysomelinae). Systematic analyses of these nodes of transitions, from no faecal recycling to faecal recycling, are needed to understand possible speciation impacts after the origin of constructions. We can surmise a shared genetic history for faecal constructions, that they have value in the survival of their builders, and they could be considered adaptive. To understand evolutionary relevance and even possible character information for phylogeny reconstruction, many more species-level studies are needed to document the life stages and to compare roles of different building materials and building repertoires.

Chrysomelid faecal-based constructions are not homologous, being formed in different ways and are held to the body by different structural modifications. Interesting points emerge when subfamily comparisons are made (Table 1). The common material of faeces points to its cheapness and ready availability. Some architectures may be convergent. Dorsal coats of faecal pellets and similar anus position in Criocerinae and in the *Blepharida*-group suggest similar neuro-physiological mechanisms (a “conveyor belt”) to move faecal pellets from anus towards the head and similar purposes. The cassidine *Eurypepla* Boheman, 1854 (Chaboo 2004) also has a wet shield, but this is built differently – the upwardly held abdomen permits the flow of viscous faeces (not pellets) down the body to coat it. It is highly likely, given findings in other non-chrysomelid debris-carriers, that specialised chaetotaxy hold the pellets onto the dorsum. The case architecture of Camptosomata—similar architecture, similar construction behaviours, and similar correlated morphologies (i.e., maternal abdominal fovea and genital ‘kotpresse’; larval flattened head, swollen abdomen, long legs, and curved claws)—support the close relationship of Cryptocephalinae and Lamprosomatinae. Comparing these aspects in the arboreal, terrestrial, and myrmecophilous species of this clade might reveal additional informative characters for taxonomy and phylogeny.

Cassidinae (e.g., Chaboo 2007) and Criocerinae (Vencl et al. 2004) both exhibit mining, cryptic and exposed larval feeders but faecal shields are made only in exposed forms; this pattern suggests these larvae use shields to protect themselves against a range of abiotic and biotic dangers that are different from those faced by their mining relatives. A bulky structure like a shield is unlikely in the constrained space of a mine.

A question in Cassidinae now is “Which tribe is the sister for the ten tortoise beetle tribes?” Borowiec (1995: fig. 2) proposed two major lineages of tortoise beetles, without identifying a particular basal tribe. Hsiao and Windsor’s topology (1995: fig. 1) resolved Spilophorini + *Oediopalpa* as phylogenetically distant from other tortoise beetles; their topology suggests either two origins of shield construction or a single origin with some losses. Chaboo (2007) found *Oediopalpa* among “hispines” and Spilophorini and Hemisphaerotini at the base of the tortoise beetle clade; this also suggests a minimum of two origins of the shield construction, yet the shields and caudal processes in these two tribes are very different. A few tortoise beetle species lack a shield, but our current phylogenetic hypotheses suggest these are secondary losses. We also know now that exuviae-only shields appear scattered over the tortoise beetle clade, suggesting multiple origins.

Two Cassidinae fossils (Chaboo and Engel 2008) support a close relationship between Notosacanthini which have mining larvae (Monteith et al. 2021) and Delocraniini which have cryptic exophagous larvae but no shield (CSC, pers. obs.). These fossils suggest that the typical tortoise beetle larval shields probably originated once and during the latest Paleocene or earliest Eocene (Chaboo and Engel 2008).

Recent field observations of *Aproida* (Aproidini) in Australia reveal that the larvae have a single caudal process and that faeces can pile up from time to time but falls off quickly: there is no fixed stable faecal shield and exuviae are not retained by larvae except at the pre-pupation stage (Chaboo, Sandoval, Campos, and Monteith unpubl. data). Leptispini have exophagous larvae that live in a cryptic leaf shelter they construct; these larvae also exhibit a single caudal process, but no shield (Prathapan et al. 2009). Species of *Eurispa* Baly, 1858 (Eurispini) have exophagous sheath-feeding larvae but the illustrations of Hawkeswood and Takizawa (1997) are unclear if they have typical caudal processes (tergal) or marginal extensions of an urogomphal plate (not homologous with caudal processes). The single caudal process appears as multiple independent origins within Cassidinae. *Discomorpha* (Omocerini) larvae exhibit a functionally single process but this appears to be a fusion of two and it retains the exuvio-faecal shield (Flowers and Chaboo 2015).

## ﻿Conclusions

We demonstrate general and widespread models of shield construction in tortoise beetles. We indicate variations in shields over the tortoise beetle clade that raise new challenges to study odd species. Many characters of shields can be defined to benefit phylogeny reconstruction, including construction repertoire, architecture, materials, and associated morphology. Natural history studies and specimen collections can integrate more species to achieve finer-scaled phylogenies of Cassidinae, particularly around nodes of transitions (e.g., mining to exophagy; presence/absence of caudal processes; presence/absence of shields). Clarifying these nodes will help us understand how life history and shields affected diversification within Cassidinae.

Defecation ecology is an important yet under-researched area that is intertwined with the building behaviours and morphology of chrysomelid beetles. Their constructions are crucial for their survival and represent adaptive macro-evolutionary events. Comparative and inter-disciplinary studies of construction behaviours are needed to better understand the evolution of chrysomelids. Until now, explanations of chrysomelid hyperdiversity have relied on the association and radiation with plants. Yet, constructions are a pervasive feature that may help explain the great subfamilial unevenness in Chrysomelidae. The major challenge is fieldwork and specimen assembly of juvenile stages and their constructions, as they are poorly represented in museum collections.

## ﻿Additional information

### Author ORCIDs

Caroline Simmrita Chaboo https://orcid.org/0000-0002-6983-8042

Sally Adam https://orcid.org/0009-0002-4971-4488

Kenji Nishida https://orcid.org/0000-0002-1846-9195

Luke Schletzbaum https://orcid.org/0000-0001-7995-7136

### Data availability

All of the data that support the findings of this study are available in the main text or Supplementary Information.

## Supplementary materials

Supplementary material 1 Film 1: *Cassidasphaerula* (Chrysomelidae, Cassidinae, Cassidinae) Data type Video (wmv file) Explanation note Larva moving shield over dorsum (1.21 mins; real-time speed; Sally Adam). YouTube link: https://youtu.be/bDyqjys6M-0. https://binary.pensoft.net/file/898803This dataset is made available under the Open Database License (http://opendatacommons.org/licenses/odbl/1.0/). The Open Database License (ODbL) is a license agreement intended to allow users to freely share, modify, and use this Dataset while maintaining this same freedom for others, provided that the original source and author(s) are credited.Caroline Simmrita Chaboo, Sally Adam, Kenji Nishida, Luke Schletzbaum

Supplementary material 2 Film 2: *Cassidasphaerula* (Chrysomelidae, Cassidinae, Cassidinae) Data type Video (wmv file) Explanation note Telescopic anus of larva excreting wet droplet (3.08 mins; real-time speed; Sally Adam). YouTube link: https://youtu.be/3vNZN60IRM8. https://binary.pensoft.net/file/898804This dataset is made available under the Open Database License (http://opendatacommons.org/licenses/odbl/1.0/). The Open Database License (ODbL) is a license agreement intended to allow users to freely share, modify, and use this Dataset while maintaining this same freedom for others, provided that the original source and author(s) are credited.Caroline Simmrita Chaboo, Sally Adam, Kenji Nishida, Luke Schletzbaum
